# A Sensing Role of the Glutamine Synthetase in the Nitrogen Regulation Network in *Fusarium fujikuroi*

**DOI:** 10.1371/journal.pone.0080740

**Published:** 2013-11-15

**Authors:** Dominik Wagner, Philipp Wiemann, Kathleen Huß, Ulrike Brandt, André Fleißner, Bettina Tudzynski

**Affiliations:** 1 Institut für Biologie und Biotechnologie der Pflanzen, Westfälische Wilhelms-Universität Münster, Münster, Germany; 2 Institut für Genetik, Technische Universität Braunschweig, Braunschweig, Germany; Georg-August-University of Göttingen Institute of Microbiology & Genetics, Germany

## Abstract

In the plant pathogenic ascomycete *Fusarium fujikuroi* the synthesis of several economically important secondary metabolites (SM) depends on the nitrogen status of the cells. Of these SMs, gibberellin and bikaverin synthesis is subject to nitrogen catabolite repression (NCR) and is therefore only executed under nitrogen starvation conditions. How the signal of available nitrogen quantity and quality is sensed and transmitted to transcription factors is largely unknown. Earlier work revealed an essential regulatory role of the glutamine synthetase (GS) in the nitrogen regulation network and secondary metabolism as its deletion resulted in total loss of SM gene expression. Here we present extensive gene regulation studies of the wild type, the Δ*gln1* mutant and complementation strains of the *gln1* deletion mutant expressing heterologous GS-encoding genes of prokaryotic and eukaryotic origin or 14 different *F. fujikuroi gln1* copies with site-directed mutations. All strains were grown under different nitrogen conditions and characterized regarding growth, expression of NCR-responsive genes and biosynthesis of SM. We provide evidence for distinct roles of the GS in sensing and transducing the signals to NCR-responsive genes. Three site directed mutations partially restored secondary metabolism and GS-dependent gene expression, but not glutamine formation, demonstrating for the first time that the catalytic and regulatory roles of GS can be separated. The distinct mutant phenotypes show that the GS (1) participates in NH_4_^+^-sensing and transducing the signal towards NCR-responsive transcription factors and their subsequent target genes; (2) affects carbon catabolism and (3) activates the expression of a distinct set of non-NCR GS-dependent genes. These novel insights into the regulatory role of the GS provide fascinating perspectives for elucidating regulatory roles of GS proteins of different organism in general.

## Introduction

 The glutamine synthetase (GS) is the only enzyme in living organisms that synthesizes glutamine as the principal nitrogen source for protein and nucleic acid biosynthesis. There are only two routes of ammonium assimilation in living cells: (1) NADP-dependent glutamate synthesis, which is catalyzed by anabolically active glutamate dehydrogenases, and (2) assimilation of ammonium by the GS. This ATP-dependent reaction involves the initial formation of γ-glutamyl phosphate from glutamate, which reacts with ammonia forming glutamine and inorganic phosphate [1,2]. 

 There are three distinct GS enzyme families that can easily be distinguished by length: GSI with 360, GSII with 450 and GSIII with 730 amino acids on average, respectively [3]. All of them form multimeric proteins containing double-ringed quaternary structures composed of identical units: GSI- and GSIII-type enzymes contain 12 identical subunits, whereas GSII- enzymes contain 10 identical subunits consisting of 2 pentameric rings [3-6]. Recent molecular studies and genome projects have shown that the genes of GSI, GSII and GSIII families are broadly distributed among prokaryotes and eukaryotes suggesting that the GS family members arose prior to the divergence of prokaryotes and eukaryotes [7-13]. Thus, GSI enzymes, which were previously thought to be limited to bacteria, have recently been identified in mammals and plants [13]. In addition to the GSI family members, representatives of the GSII family, which were thought to be exclusive to eukaryotes, have been found in all screened *Streptomyces*, *Agrobacterium*, *Rhizobium* and *Frankia* spp. strains [14]. However, in filamentous fungi only GSII family proteins were identified so far, and in most cases only one GS-encoding gene is present in the fungal genomes. Interestingly, in the filamentous fungus *Neurospora crassa* two non-identical subunits of the GSII family, GSα and GSβ, have been identified and *in vitro* translation indicated that different mRNAs code for GSα and GSβ subunits [15-17]. The presence of two GS-encoding genes was later confirmed by genome sequencing [18].

 Since glutamine is a key metabolite in nitrogen metabolism both the intracellular glutamine pool as well as the activity of GS are tightly regulated. Based on experimental data in various fungi it became widely accepted that glutamine is a (if not “the”) key effector of nitrogen catabolite repression (NCR), a regulatory circuit that ensures the preferential utilization of reduced nitrogen sources such as ammonium and glutamine over more complex and energy-demanding ones, e.g. nitrate, purines and proteins [19-26]. However, beside glutamine also ammonium, glutamate and nitrate might be sensed by specific sensors and generate signals for nitrogen metabolite repression [24,25,27,28].

 Seminal work in *Saccharomyces cerevisiae* established a model where the target of rapamycin (Tor) complex kinase 1 (TorC1) senses intracellular glutamine provided by the GS, thereby transmitting the signal of glutamine availability to the GATA-type transcription factors Gln3 and Gat1. These GATA factors activate transcription of NCR-sensitive genes only under nitrogen-limiting conditions or in the presence of a non-preferred nitrogen source [29]. Recently, this linear model of signal transduction has been challenged. The addition of rapamycin (inhibiting TorC1) had a different effect on Gln3 and Gat1 (the homologs of AreA and AreB in *Fusarium fujikuroi*) localization, their DNA-binding capabilities and NCR-sensitive gene expression, than a treatment with l-methionine-*S*-sulfoximine (MSX; inhibiting GS enzymatic activity). These data suggest the existence of two parallel nitrogen-responsive signalling pathways in yeast, one dependent on TorC1, and the other one dependent on the GS [30]. However, the question of how the GS (MSX)-responsive signal is wired to the level of NCR-sensitive genes in *S. cerevisiae* and filamentous fungi remains unresolved.

 In prokaryotes, a direct role of GS in nitrogen regulation is well established. In *B. subtilis*, GSI activity is feedback-inhibited by glutamine [31], and this inhibited form of the GS modulates the DNA-binding capabilities of the two transcription factors TnrA and GlnR by direct protein-protein interaction [31,32]. Both transcription factors regulate gene expression in response to changes in nitrogen availability. In *Escherichia coli*, GSI is subject to cumulative feedback inhibition by glutamine and end products of glutamine metabolism, such as ADP, AMP and other nucleotides by competing with the substrate glutamate for the active site [33].

 Although a direct interaction of GS enzymes with potential transcription factors was never demonstrated in any eukaryotic organism, it has been proposed that the GS might be the key regulator in the nitrogen regulation network also in higher organisms. In *N. crassa*, two allelic *gln1* gene mutants*, gln1a* and *gln1b*, have been generated in the 1980’s and were shown to have derepressed levels of nitrate and nitrite reductase activity in the presence of ammonium, glutamate, and/or glutamine, suggesting that the GS may potentially be involved in sensing the nitrogen status and signaling to downstream effectors [21,22]. However, the nature of these mutations in the two GS-encoding genes was never elucidated.

 The rice pathogenic fungus *F. fujikuroi* is well known for its production of a family of plant hormones, the gibberellic acids (GAs) [34]. Beside GAs, the fungus produces a set of other economically important SMs, such as the red pigments bikaverin [35] and fusarubins [36-38], as well as mycotoxins such as fusarins, fusaric acid, beauvericin and fumonisins [38-42]. The biosynthesis of GAs and bikaverin are repressed by high nitrogen concentrations by different molecular mechanisms. GAs were the first SMs for which a strict dependency on the major nitrogen regulators, the GATA transcription factor AreA and AreB has been shown [43,44,49] (unpublished data). In contrast, bikaverin is subject to a non-canonical AreA-independent nitrogen regulation [35]. Beside AreA and AreB, other regulators, such as the AreA-binding protein Nmr [43-45] and the bZIP transcription factor MeaB [46] are also involved in regulation of SM production in *F. fujikuroi*.

 Previously we deleted the GS-encoding gene, *gln1* in order to increase GA production by lowering the intracellular level of glutamine. However, in contrast to our expectation, deletion of *gln1* or addition of MSX did not result in upregulation, but in repression of GA and also of bikaverin biosynthetic genes, suggesting a regulatory function of the GS in *F. fujikuroi* [47]. Beside SM biosynthetic genes, several other target genes of the GS were identified by differential cDNA screening between the wild type and the *gln1* mutant, e.g. those encoding the isocitrate lyase, the uricase, translation initiation and elongation factors, and two small proteins with unknown function, DDR48 and CipC [47]. Our first speculation was that the cross pathway control regulator Cpc1, which is known to sense imbalances in amino acid pools, is involved in loss of SMs in response to glutamine limitation. However, this possibility was ruled out: targeted deletion of *cpc1* did not alter the down-regulation of GA and bikaverin biosynthetic genes when the GS was inactivated by MSX [48].

 The aim of this work is to deepen our knowledge about the role the GS plays in the nitrogen regulation network in *F. fujikuroi*. Our working hypothesis was that the GS exerts a regulatory function independently of its glutamine-providing enzymatic activity. To test this hypothesis, we complemented the *F. fujikuroi* Δ*gln1* mutant 1) with different fungal (wild-type and mutant GS-encoding genes of *N. crassa*) and prokaryotic (GSI and GSII of *Streptomyces coelicolor*) GS-encoding genes, and 2) with *gln1* gene copies of *F. fujikuroi* carrying point mutations in all 14 highly conserved sequence domains. The complemented strains generated by both approaches were monitored for their dependency on glutamine (glutamine auxotrophy/prototrophy) and their ability to express NCR-sensitive secondary and primary metabolism genes. Collectively, our data support the existence of a GS-dependent nitrogen-responsive pathway for SM biosynthetic genes that is independent of the glutamine-providing enzymatic function.

## Materials and Methods

### Fungal strains and culture conditions

 The following *F. fujikuroi* strains were employed: wild-type strain IMI58289 (Commonwealth Mycological Institute, Kew, UK), the *gln1* deletion strain Δ*gln1-*T41 [47], the *areA* deletion strain, Δ*areA*-T19 [49], the *nmr* deletion strain Δ*nmr*-T20 [43], and the *meaB* deletion strain Δ*meaB-*T10.3 [46].

 The *N. crassa* wild-type, the Δ*gln1* mutant strains FGSC1449 (mutant *gln-1a*) and FGSC4536 (mutant *gln-1b*) [20,21] were provided by the Fungal Genetics Stock Center. *N. crassa* knockout strains of both mating types, carrying deletions in one of the two GS-encoding genes (NCU_04856: FGSC_18811, FGSC_18812 and NCU_06724: FGSC_19958, FGSC_119959) were obtained from the *Neurospora* Functional Genomics Project (http://www.dartmouth.edu/~neurosporagenome/).

*F. fujikuroi* strains were precultivated for 72 h in 300 ml Erlenmeyer flasks with 100 ml Darken medium [50] with 18 mM glutamine, on a rotary shaker at 28°C on a rotary shaker at 200 rpm for 3 days. 500 μl of this culture was used as inoculum for cultivations in ICI media (Imperial Chemical Industries Ltd., UK) [51] with 6 mM (10 % ICI medium) or 60 mM (100% ICI medium) glutamine. For DNA isolation and protoplasting, *F*. *fujikuroi* strains were incubated in 100 ml modified ICI medium (Imperial Chemical Industries Ltd., UK) [51] containing 10 g/l fructose and additional 18 mM glutamine at 28°C on a rotary shaker at 200 rpm for 3 days or 18 h, respectively. For RNA isolation, the mycelia were harvested after addition of indicated nitrogen sources and time points (see text). For analysis of SMs, the the strains were grown in ICI medium with 6 mM or 60 mM glutamine.

### Bacterial strains and plasmids

*E. coli* strain TOP10 (Invitrogen, Groningen, The Netherlands) was used for plasmid propagation. Glutamine synthetase gene copies from *F. fujikuroi*, *N. crassa* and *S. coelicolor* were cloned by PCR using primers with ApaI and SalI restriction sites (Table S1) and cloned adjacent to the *F. fujikuroi gln1* promoter in pUCH-N-*gln1*_prom_ [48,52] carrying the nourseothricin resistance cassette. Plasmids pLHAp and pLHIIp, carrying the *S. coelicolor gln1* and *glnII* genes, respectively, were kindly provided by W. Wohlleben (University Tübingen, Germany).

### Nucleic acid isolation and sequence analysis

 Lyophilized mycelium was ground into a fine powder and dispersed (in the case of DNA for use in PCR) in extraction buffer as described by Cenis [53]. DNA for Southern hybridization experiments was prepared following the protocol of Doyle and Doyle [54]. Plasmid DNA was extracted using the Genomed plasmid extraction kit (Genomed, Germany). Total *F. fujikuroi* RNA was isolated using the RNAgents total RNA isolation kit (Promega, Mannheim, Germany). Samples of 20 µg of total RNA were transferred to Hybond-N^+^ membranes after electrophoresis on a 1% (w/v) agarose gel containing 1% (v/v) formaldehyde, according to Sambrook et al. [55]. Northern blot hybridizations were accomplished by the method of Church and Gilbert [56]. For Southern analysis, genomic DNA was digested with appropriate restriction enzymes, fractionated in 1 % (w/v) agarose gels, and transferred to nylon membranes. DNA probes were randomly labelled using P^32^ oligonucleotides and hybridizations were carried out overnight at 65°C. PCR reactions contained 25 ng DNA, 5 pmol of each primer, 200 nM desoxynucleotide triphosphates, and 1 unit BioTherm DNA polymerase (GeneCraft GmbH, Lüdinghausen, Germany). The reactions were started with 4 min at 94 °C, followed by 35 cycles of 1 min at 94 °C, 1 min at 56°C to 65°C, 1 min at 70°C, and a final 10 min at 70°C. DNA and protein sequence alignments and phylograms were done with DNA STAR (Madison, WI, USA). Sequence homology searches were performed using the NCBI database server. Protein homology was based on BlastX searches [57].

### PCR

 All primers used for PCR were obtained from Biolegio (Netherlands) (Table S1). PCR reactions contained 25 ng DNA, 5 pmol of each primer, 200 nm dNTPs, and 1 unit of BioTherm^TM^DNA polymerase (GeneCraft GmbH, Lüdinghausen, Germany) and were initiated with a 4 min soak at 94 °C followed by 36 cycles of 1 min at 94 °C, 1 min at 56 to 65 °C, 1-3 min at 70 °C, and a final soak for 10 min at 70 °C. PCR products were cloned into pCR^®^2.1‑TOPO^®^ vector using the TOPO TA Cloning^®^ kit (Invitrogen, Groningen, The Netherlands) and transformed into *Escherichia coli* (Invitrogen). Plasmid DNA from *E. coli* was extracted using the GeneJET^TM^ Plasmid Miniprep Kit (Fermentas GmbH, St. Leon-Rot, Germany) and sequenced using the BigDye^®^ Terminator v3.1 Cycle Sequencing Kit and the ABI Prism® 3730 Genetic Analyzer (Applied Biosystems, Foster City, CA, USA) according the manufacturer’s instructions.

### Site directed mutagenesis and generation of knock-out mutants

 Site-directed mutagenesis was carried out as described by the manufacturer using the QuikChange® II Site-Directed Mutagenesis Kit (Agilent Technologies). To generate a template vector for site directed mutagenesis, the full length *gln1* cDNA fragment from *F. fujikuroi* was amplified from cDNA by using the RT-primers *gln1*-F-SalI and *gln1*-R-ApaI (Table S1), and the PCR fragment was cloned into vector pCR^®^2.1-TOPO^®^ (Invitrogen) resulting in vector p*gln1*-cDNA. Primers for introducing specific point mutations into the wild-type *gln1* cDNA sequence are listed in Table S1. The mutated gene copies were then cut out with ApaI and SalI and cloned into the destination vector pUCH-N-*gln1*_prom_ [44] and subsequently transformed into mutant ∆*gln1* [47]. For gene replacements, the plasmids pΔniaD and pΔniaD were assembled using yeast recombinational cloning as essentially described for *N. crassa* deletion vectors [58] and recently established for *F. fujikuroi* vectors [59]. The 5’ and 3’ flanks of *niaD* and *niiA* were amplified using primer pairs ”gene”-5’-F1/-R1 and ”gene”-3’-F1/-R1, respectively. Plasmid DNA from *S. cerevisiae* was extracted using the GeneJET^TM^ Plasmid Miniprep Kit (Fermentas GmbH, St. Leon-Rot, Germany) with slight modifications: cells were resuspended in 300 µL Resuspension solution plus 100 µl glass beads, lyzed by addition of 600 µl Lysis Solution and neutralized with 450 µl Neutralization Solution. 

### Fungal transformations

 Preparation of protoplasts of *F. fujikuroi* was carried out as described [60]. 10^7^ protoplasts of strain Δ*gln1*-T41 were transformed with 10 µg of the pUCH-N-*gln1*_prom_ vector [44] carrying the wild-type or a mutant gene copies of GS-encoding genes from *F. fujikuroi*, *N. crassa*, and *S. coelicolor*, repsectively. The transformed protoplasts were regenerated at 28 °C in a complete regeneration agar (0.7 M sucrose, 0.05 % yeast extract) containing 100 µg/ml nourseothricin (Werner Agents, Jena, Germany) for 6 - 7 days.

### *N. crassa* transformations and crosses

 Assembly of the gene replacement cassette and transformation of *N. crassa* were performed according the Gene Knock-out Protocol of the *Neurospora* Functional Genomics Project (http://www.dartmouth.edu/~neurosporagenome/). Crosses were performed as described in [61].

### Analysis of secondary metabolites

 For analysis of gibberellin formation, the wild type strain, the Δ*gln1* mutant and all transformants expressing heterologous or mutated gene copies were cultivated in ICI medium with 6 mM glutamine for 5 days. GAs were analyzed by HPLC according to Barendse and van de Werken [62] using a Merck HPLC system with a UV detector and a Lichrospher 100 RP18 column (5μm; 250x4; Merck). 

Bikaverin was analyzed in the same cultures as GAs by HPLC [35]. 

### Plate assays

 The growth of the transformants complemented with one of the GS-encoding genes from different microorganisms or mutated copies of *F. fujikuroi gln1* was compared with that of the *F. fujikuroi* wild-type and the recipient strain, the Δ*gln1* mutant, on complete medium (CM) [63] with additional 18 mM of glutamine as nitrogen source and minimal Czapek-Dox medium containing NaNO_3_ as nitrogen source. Plates were incubated at 28° C for three days in the dark. Solidified ICI complemented with 1 mM glutamine and either 9 mM glutamine, 4.5 mM ammonium tartrate, 9 mM sodium nitrate or 9 mM glutamate was used for growth assays of the wild type, the Δ*gln1* and Δ*niaD* mutants. 

 To compare growth and development of the *N. crassa* wild-type with the single and double Δ*gln* mutants, strains were grown on Vogel’s minimal medium [64] without glutamine or supplemented with 27 mM glutamine. Slant tube cultures were incubated at 30°C, race tubes at room temperature.

### Western blot analyses

 Total protein extraction was performed as described in [65]. 50 µg of the protein extract were used per lane and separated by discontinuous SDS-polyarcylamide gel electrophoresis. The 5% loading gel was used at a pH value of 6.8 while the 10% separation gel had a pH value of 8.8. The resulting gel was electro-blotted (semi-dry) to a nitrocellulose transfer membrane. For detection of the *F. fujikuroi* GS, polyclonal anti-TbGS antibodies (1:5000 dilution) were used [23]. To detect the primary antibodies HRP (Horse radish peroxidase) conjugated anti-rabbit secondary antibodies were used in a 1:10000 dilution, followed by visualization of the occurring chemoluminescence. 

### Determination of intracellular free amino acid concentrations

 Lyophilized mycelium of the appropriate cultivations was ground into a fine powder and extracted according to the protocol in [66]. The re-suspended amino acids were derivatized with o-phthalaldehyde and analyzed by RP-HPLC according to a published procedure [67]. The cell debris remaining after the four extractions was dried to constant weight at 80°C and the dry weight was determined.

## Results

### Regulation of gln1 expression and GS protein levels by AreA, MeaB and Nmr

 To intensify our understanding about regulation of GS activity, we first compared the *gln1* transcript and GS protein levels in the wild type with those in three regulatory mutants harboring targeted deletions of the highly conserved AreA-, Nmr1, and MeaB-encoding genes [43-46,68,69], respectively. 

**Figure 1:**
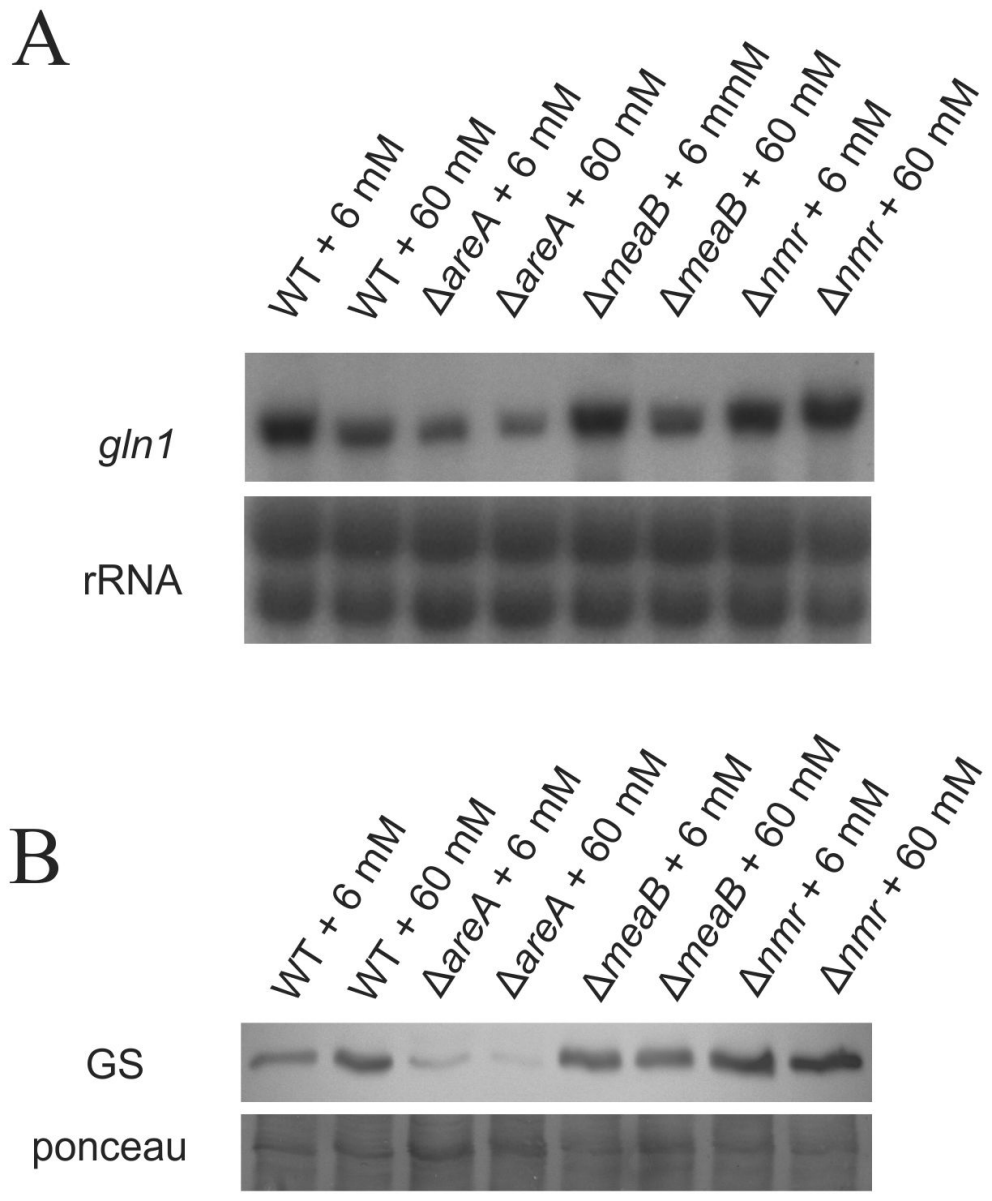
The *gln1* gene expression and GS protein levels are positively regulated by AreA. Regulation of *gln1* gene expression and GS protein levels in *F*. *fujikuroi*. **A**. The wild-type (WT), Δ*areA*, Δ*meaB* and Δ*nmr* strains were grown for 3 days in ICI submerse culture with either 6 mM or 60 mM glutamine as nitrogen source. Total RNA was used for northern analysis using the genomic gln1 fragment as probe. *18S* rRNA was visualized as a loading control. **B**. Total protein extract was used for western analysis using the polyclonal anti-TbGS antibodies and HRP-conjugated anti-rabbit secondary antibodies. Ponceau staining as loading control.

 We studied *gln1* transcript and GS protein levels under both nitrogen-limiting (6 mM glutamine) and nitrogen-sufficient (60 mM glutamine) conditions. In the wild type as well as ∆*nmr1* and ∆*meaB* mutants the *gln1* transcript levels were high under both conditions, with slightly stronger expression under nitrogen starvation conditions. In contrast, expression of *gln1* is slightly reduced in the ∆*areA* mutant independently of the glutamine concentration (Figure 1 A).

 Western blot analysis correlated well with the results of transcript studies regarding the role of AreA in regulating GS activity. The protein level of the GS was significantly reduced in the *∆areA* mutant compared to the other strains. It is noteworthy that the highest GS levels were found in the *∆nmr* mutant, probably due to higher activity of AreA (Figure 1B). In summary, the *gln1* transcript and GS protein levels are positively regulated by AreA, while Nmr and MeaB exert weak antagonistic functions. 

### The GS affects expression of nitrogen regulatory and secondary metabolite genes

 Since we observed a strong down-regulation of GA biosynthetic genes in the *gln1* deletion mutant in earlier studies [47], we wanted to investigate if this down-regulation is due to altered expression of nitrogen regulatory genes, such as *areA*, *areB*, and *nmr*, in the ∆*gln1* mutant. Alteration of *areA* expression levels could be an explanation for the significantly decreased expression of AreA-dependent GA biosynthetic genes (e.g. *cps/ks* encoding the *ent*-copalyl/*ent*-kaurene synthase), but not for down-regulation of AreA-independent bikaverin biosynthetic genes [35,44,49]. 

**Figure 2:**
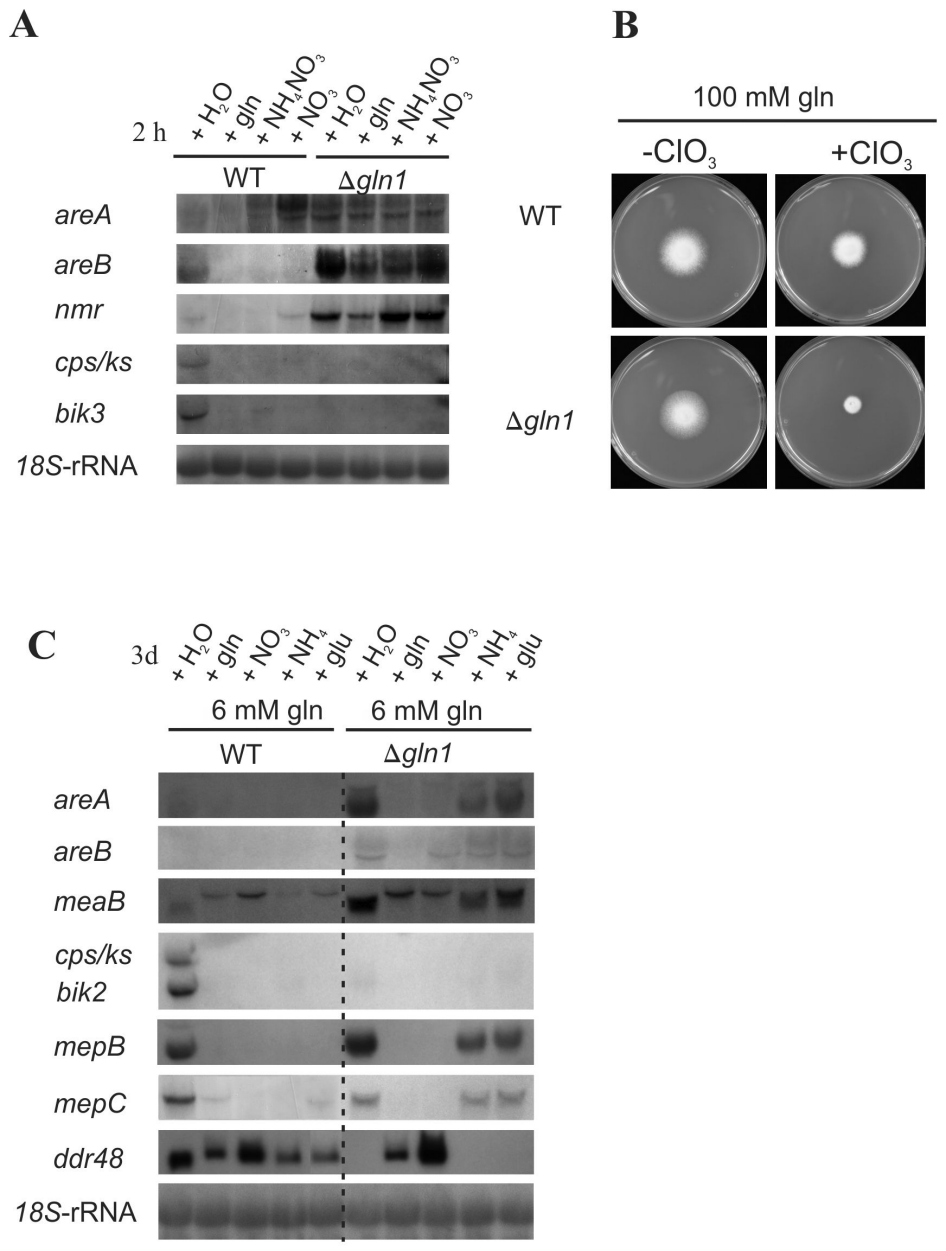
GS-dependent and independent sensing of different nitrogen sources. Transcription of NCR-sensitive and -insensitive genes in response to different nitrogen sources in the wild type and the Δ*gln1* strain. **A**. The wild type (WT) and the Δ*gln1* strain were grown for 3 days in ICI submerse cultures with 6 mM glutamine as nitrogen source. After 3 days glutamine (gln), NH_4_NO_3_ or NaNO_3_ (NO_3_^-^) were added to a total concentration of 60 mM in case of gln and NH_4_NO_3_, and 120 mM in case of NaNO_3_. An equal volume of H_2_O was added as control. The mycelia were harvested 2 hours after nitrogen addition (short term exposure), and total RNA was used for northern analysis. *18S* rRNA was visualized as a loading control. **B**. The wild-type (WT) and the Δ*gln1* mutant were grown for five days on CM agar with 100 mM glutamine and with or without 50 mM KClO_3_. If AreA is active the nitrate reductase-encoding gene *niaD* is expressed, and the nitrate reductase reduces KClO_3_ to the toxic KClO_2_. In the wild type, high glutamine levels repress the expression of *areA* and *niaD*, while the growth of the Δ*gln1* mutant is restricted due to an active AreA and subsequent expression of *niaD* leading to accumulation of toxic KClO_2_. **C**. The wild-type (WT) and the Δ*gln1* strains were grown for 3 days (long term exposure) in ICI submerse cultures with 6 mM glutamine (control; only water was added) or 6 mM glutamine and additionally 54 mM glutamine (gln), 54 mM ammonium tartrate (NH_4_^+^), 108 mM NaNO_3_ (NO_3_^-^) or 108 mM glutamate (Glu) as nitrogen source. Total RNA (15 µg) was used for northern analysis and hybridized with probes as indicated. *18S* rRNA was visulaized as a loading control. Abbreviations: *mepB* and *mepC*, ammonium transporter genes.

 The wild type and the *∆gln1* strains were grown in submerged cultures with low levels of glutamine (6 mM). After 72 hours of incubation, when glutamine is exhausted, we added either no nitrogen (nitrogen starvation) or one of the three nitrogen sources: glutamine, ammonium nitrate (each 60 mM) or sodium nitrate (120 mM) and incubated the mycelia for two more hours in order to study the immediate response to nitrogen addition (short term effect). In the wild type, *areA*, *areB* and *nmr* genes are expressed under nitrogen starvation conditions. Additionally, *areA* is well expressed in the presence of nitrate, probably due to its role in activating of nitrate utilizing genes as shown in *Aspergillus* [70-72]. In contrast, glutamine and ammonium represses the transcription of all three genes. Surprisingly, *areA*, *areB* and *nmr* are significantly upregulated in the *∆gln1* mutant compared to the wild type under all conditions tested (Figure 2A). These results indicate that the ∆*gln1* mutant is unable to sense the exogenously added nitrogen sources under these experimental conditions resulting in enhanced expression of these otherwise repressed genes. However, despite the enhanced expression of *areA* and *areB* in the ∆*gln1* mutant, expression of the AreA- and AreB-dependent GA biosynthetic gene *cps/ks* was abolished, indicating that either AreA and/or AreB are nonfunctional, or that the GS itself is important for *cps/ks* expression. Since *nmr*, an AreA target gene in *F. fujikuroi* [44], was highly expressed in the ∆*gln1* mutant non-functionality of AreA seems unlikely (Figure 2A). To substantiate this hypothesis, we grew both the wild type and ∆*gln1* mutant on medium containing the nitrate analogon KClO_3_. The *gln1* mutant showed a much more restricted growth on KClO_3_ compared to the wild type, indicating enhanced activity of AreA and the AreA-dependent nitrate reductase, resulting in an elevated conversion of KClO_3_ to the toxic KClO_2_ by nitrate reductase activity (Figure 2B). 

 To gain a deeper insight into the role of GS in nitrogen sensing and signaling, we compared the effects of different nitrogen sources on the expression of NCR sensitive genes such as the ammonium permease-encoding genes (*mepB* and *mepC*) [73], SM biosynthetic genes (*cps/ks* and *bik2*), and the regulatory genes *areA*, a*reB*, and *meaB* under long term (three days) incubation conditions. The wild type and the *gln1* mutant were grown for three days in synthetic medium with either 6 mM glutamine (nitrogen-limiting conditions) or in the same medium with additional glutamine, glutamate, ammonium tartrate, or sodium nitrate to an equal final nitrogen concentration of 120 mM (nitrogen surplus conditions). Similar to our previous observations for short term response of nitrogen addition (two hours), *areA* and *areB* transcript levels were up-regulated in the *∆gln1* mutant after 3 days incubation, but only under nitrogen-limiting conditions (6 mM glutamine) or if one of the two GS substrates, glutamate or NH_4_^+^, was present in addition to 6 mM glutamine (Figure 2C). Also *meaB*, encoding a nitrogen-responsive bZIP trancription factor [46] is upregulated in the *gln1* mutant compared the wild type. Previously, we have shown that *meaB* gives rise to two distinct mRNA transcripts, *meaB* ^*S*^ (expressed under nitrogen-limiting conditions in an AreA-dpendent manner) and *meaB* ^*L*^ (expressed under nitrogen sufficient conditions) [46]. In the wild-type, *meaB* ^*S*^ is detectable only under nitrogen-limiting conditions, whereas *meaB* ^*S*^ appears in the ∆*gln1* mutant also under high ammonium or glutamate concentrations (Figure 2C). These data suggest that ammonium and glutamate appear not to be sensed in the *gln1* mutant resulting in de-repression of *areA* and subsequent appearance of the *meaB* ^*S*^ transcript despite nitrogen sufficiency. 

 Several AreA-dependent genes such as *mepB* and *mepC* [73], resembled the *areA* transcription profile in the ∆*gln1* mutant: they are repressed with glutamine and nitrate, but deregulated with ammonium and glutamate (Figure 2C). In contrast, expression of genes involved in secondary metabolism such as the AreA-dependent *cps/ks* and AreA-independent *bik2* genes was lost in the ∆*gln1* mutant even under favoring nitrogen starvation conditions compared to the wild type (Figure 2C). 

 Taken together, these data indicate that there is a clear regulatory difference between NCR-sensitive genes involved in primary metabolism (e.g. *mepB* and *mepC*) and regulation (e.g. *areA*, *areB*, *meaB*), and those involved in secondary metabolism (e.g. GA and bikaverin genes). Genes involved in primary metabolism and regulation are repressed by 1) glutamine and nitrate independently of the GS, and 2) by NH_4_^+^ and glutamate in a GS-dependent manner, i.e. they are derepressed in the ∆*gln1* mutant under conditions where these nitrogen sources are present in access. In contrast, SM biosynthetic genes are not expressed in the ∆*gln1* mutant under all conditions tested underlining their strict dependency on the presence of a functional GS (Figure 2C). 

### Do heterologous GSI- and GSII-encoding genes complement enzymatic and regulatory defects in the F. *fujikuroi* Δ*gln1* mutant?

 Comparative genomic analyses between the recenty sequenced genome of *F. fujikuroi* and all other so far sequenced fungal genomes showed that several SMs produced by *F. fujikuroi*, e.g. GAs, are very specific for this species [42]. Therefore, we wanted to explore the possibility if heterologous GS-encoding genes can restore both the enzymatic activity (glutamine formation) and regulation of SM production. Therefore, we complemented the *F. fujikuroi* Δ*gln1* mutant with the two GSII-encoding genes from *N. crassa* and GSI- and GSII-encoding genes from *S. coelicolor*. Our hypothesis was that the heterologous GS-encoding genes might overcome glutamine auxotrophy, but probably not the defective *Fusarium*-specific secondary metabolism.

 For *S. coelicolor* it was shown that both *glnII* (GSII-type) and *gln1* (GSI-type) genes are fully functional independently of each other [14,74]. However, it is not yet known why *N. crassa* harbors two highly similar GS copies, encoded by the two distinct genes NCU04856.5 (*Ncgln1*) and NCU06724.5 (*Ncgln2*), and if both genes are essential to form an active GS multimer as suggested [75-77]. Growth tests on minimal medium showed that both genes encode fully functional GS proteins that can complement each other (detailed experimental informations in supplementary material, Figure S1). However, it was not possible to generate homokaryotic ΔΔ*Ncgln1*/*Ncgln2* double knock-out mutants, and even the heterokayotic mutant strains revealed growth defects on minimal medium (Figure S1). 

 The *S. coelicolor* and *N. crassa* GS-encoding genes were individually fused with the *F. fujikuroi gln1* promoter and transformed into the *F. fujikuroi* Δ*gln1* strain, respectively. Beside the wild type genes *Ncgln1* and *Ncgln2* we also examined the functionality of the *Ncgln2* gene copies from the GS-defective *N. crassa* mutants *gln-1a* and *gln-1b*, which were previously shown to have a 20- to 30-fold lower GS-activity [75,76] and to be deregulated in the presence of ammonium [20,21]. Since the nature of the mutations in these two strains (*gln-1a* and *gln-1b*) has never been studied, we sequenced *Ncgln1* and *Ncgln2* genes in both mutants and identified several point mutations in both GS-encoding genes, but mainly in the *Ncgln2* gene of both strains (Table S2). 

**Figure 3:**
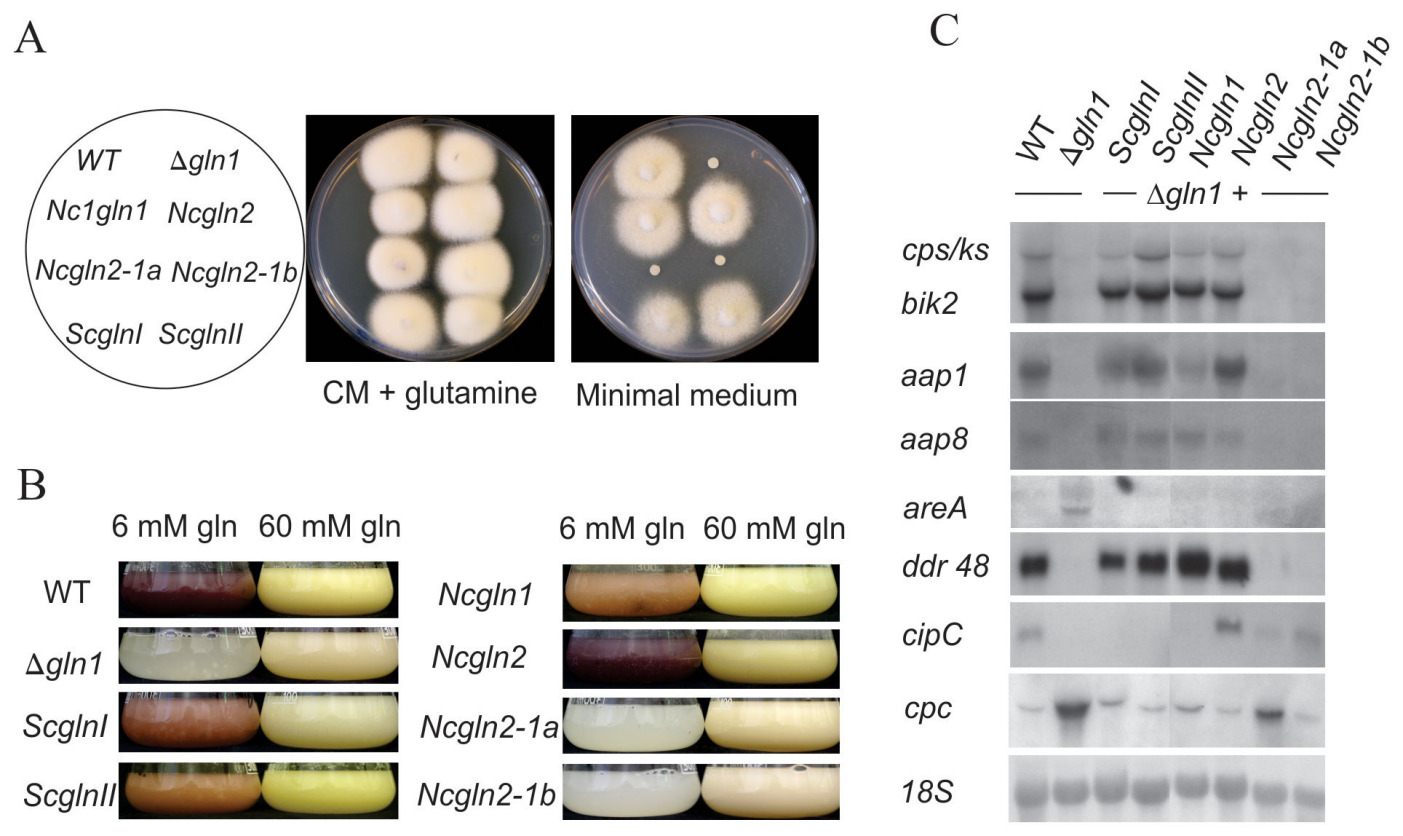
Characterization of heterologous Δ*gln1* complementation mutants. **A**. Plate assays comparing the growth of the wild type (WT), the Δ*gln1* mutantand different complemented transformants. Complete medium (CM) contained 18 mM glutamine, while the minimal medium (CD) contained no additional nitrogen source. Pictures were taken after 3 days of cultivation. **B**. Pictures of the wild type (WT), the Δ*gln1* mutant and the heterologous GS complementants grown for 3 days in ICI submerse cultures with either 6 mM glutamine (gln) (GA- and bikaverin-inducing (red coloration) conditions) or 60 mM glutamine as nitrogen source. **C**. Total RNA was isolated from mycelia grown for 5 days in ICI submerse cultures with 6 mM glutamine and used for northern blot analysis. *18S* rRNA was visualized as a loading control. Abbreviations: *aap1* and *aap8*, amino acid transporter genes; *cipC*, *ddr48* and the cross-pathway control gene *cpc* were found to be GS-target genes [47]; *mepB*, ammonium transporter gene.

 Both *N. crassa* wild type genes, *Ncgln1* and *Ncgln2*, were able to restore wild-type-like growth on minimal medium without glutamine, whereas none of the *Ncgln2* mutant alleles (*Nc2-1a* and *Nc2-1b*) did (Figure 3A), thereby substantiating our previous conclusions that both *N. crassa* wild type genes encode fully functional enzymes (Figure S1). Surprisingly, transformants carrying either the GSI- or the GSII-type GS-encoding *S. coelicolor* gene were also able to grow on minimal medium without glutamine, indicating a high level of functional conservation between GS from prokaryotic and eukaryotic organisms (Figure 3A). Furthermore, *Ncgln1*, *Ncgln2*, *Scgln1* and *ScglnII* genes were able to restore wild-type-like growth in submers culture with 6 mM glutamine (Table 1). By contrast, the defective *Nc2-1a* and *Nc2-1b* gene copies accumulated significantly less biomass similar to the Δ*gln1* mutant.

**Table 1:** Growth and secondary metabolite production of the *F. fujikuroi* wild type, the Δ*gln1* mutant and complementants transformed with prokaryotic and eukaryotic GS-encoding genes.

**Strain**	**Growth on MM (CD)**	**DW in submers culture with 6 mM gln**	**Production of bikaverins**	**Production of gibberellins**
*Fusariumfujikuroi* wild type	**Yes**	1.38	**yes**	**yes**
*Ff*Δ*gln1*	No	0.19	no	no
*Ff*Δ*gln1* + *Str. coelicolor gln1*	**Yes**	0.89	**yes**	**yes**
*Ff*Δ*gln1* + *Str. coelicolorglnII*	**Yes**	1.20	**yes**	**yes**
*Ff*Δ*gln1* + *N. crassa gln1*	**Yes**	0.99	**yes**	**yes**
*Ff*Δ*gln1* + *N. crassa gln2*	**Yes**	1.25	**yes**	**yes**
*Ff*Δ*gln1* + *N. crassa gln2-1a*	No	0.20	no	n.a.
*Ff*Δ*gln1* + *N. crassa gln2-1b*	No	0.18	no	n.a.

n.a. = not tested; DW- dry weight (g/100 ml) after 5days growth in minimal synthetic medium with 6 mM glutamine; MM- minimal medium

 To show if restoration of enzymatic GS activity and wild type-like growth by heterologous GS-encoding genes correlates with restoration of secondary metabolism, and if missing enzymatic activity of the *Nc2-1a* and *Nc2-1b* gene alleles correlates with the lack of secondary metabolism, GA and bikaverin production were analyzed in all strains after five days growth under inducing conditions (6 mM glutamine). The data obtained show a strong correlation between glutamine prototrophy and the ability to produce these SMs (Table 1). The two *N. crassa* wild-type genes *Ncgln1* and *Ncgln2* and the two *Streptomyces* genes, *Scgln1* and *ScglnII*, which could restore wild-type growth of the Δ*gln1* mutant on minimal medium (Figure 3A), were also able to restore bikaverin and GA gene expression and concomitant production of the chemical compounds (Figure 3B and C; Table 1).

 All transformants carrying an active GS enzyme revealed wild-type-like expression levels for canonical AreA target genes such as the amino acid permease-encoding genes *aap1* and *aap8* (Figure 3C). By contrast, *aap1* and *aap8* are not expressed in the Δ*gln1* mutant and in transformants containing non-functional *Ncgln2* gene copies (*Ncgln2-1a*; *Ncgln2-1b*) from *N. crassa* mutant strains gln-1a and gln-1b (Figure 3C).

 Furthermore, we studied the correlation between GS enzyme activity and expression of the previously identified GS target genes *ddr48*, *cipC* and *cpc* [47]. Similar to the amino acid permease-encoding genes, expression of *ddr48* was only restored in transformants carrying one of the wild type *N. crassa* or *Streptomyces* GS-encoding genes. An unexpected expression pattern was observed for *cipC*, a nitrogen-independent GS target gene [47]. The *cipC* gene family is unique to the fungal kingdom and is reported to be expressed during the adaption to pathogenic growth in *Aspergillus fumigatus* and *Ustilago maydis* [78,79]. Unlike all other tested genes, expression of *cipC* could not be restored by the two functional GS-encoding genes from *S. coelicolor* or *Ncgln1* from *N. crassa*. Only *Ncgln2* and, surprisingly, its mutated, non-functional copies *Ncgln2-1a* and *Ncgln2-1b* were able to restore *cipC* expression. Similarly, the lower wild-type-like expression of the GS target gene *cpc* encoding the cross-pathway control transcription factor FfCpc [47,48] was also restored by the non-functional mutant copy of *Ncgln2-1b* and partially by *Ncgln2-1a* (Figure 3C). 

 In summary, GS-encoding wild type genes (GSI and GSII-type) of *N. crassa* and *S. coelicolor* restored wild-type-like growth on minimal solidified medium (prototrophy), wild type-like biomass formation in submers culture with 6 mM glutamine, and the wild-type-like expression pattern of NCR sensitive genes involved in primary (*aap1*, *aap8*) and secondary metabolism (GAs, bikaverin). A separation of enzymatic activity and regulatory function of the GS was obtained only regarding expression of the two GS-dependent genes *cipC* and *cpc*, which were also expressed in strains complemented with non-functional *Ncgln2* gene copies. The partial restoration of *cipC* and *cpc* by non-functional GS proteins is a first indication for a potential regulatory role of the GS protein itself.

### Site-directed mutagenesis of the F. *fujikuroi* gln1 gene

**Figure 4:**
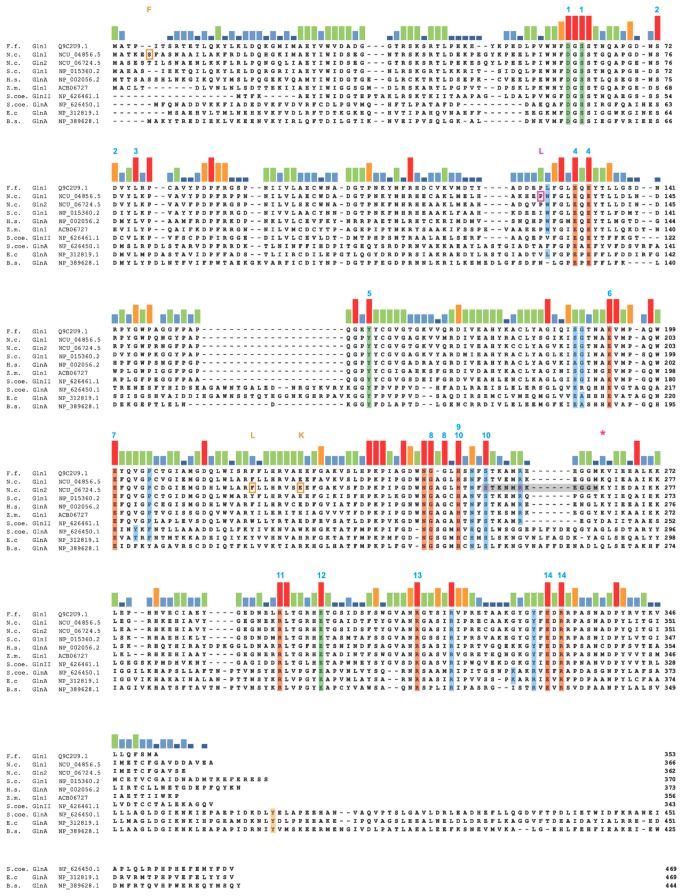
ClustalW alignment reveals conserved regions in fungal GS proteins. ClustalW alignment of indicated GS proteins with corresponding NCBI identifiers (or Broad Institute identifiers for *N*. *crassa* sequences): F.f = *F*. *fujikuroi*, N.c. = *N*. *crassa*, S.c. = *S*. *cerevisiae*, H.s. = *H*. *sapiens*, Z.m. = *Z*. *mays*, S.coe. = *S*. *coelicolor*, E.c. = *E*. *coli*, B.s. = *B*. *subtilis*. Colored bars represent levels residue conservation (red = high; dark blue = low). Residues in green mark putative NH_4_^+^ binding sites, residues in orange mark putative glutamate binding sites, residues in blue mark putative ATP binding sites and residues in yellow indicate adenylylation site of GSI proteins. Residues in grey indicate site of missing base pair in NcGln2 of the *N*. *crassa* *gln-1a* mutant and subsequent sequence change to FPPRTCARRVA followed by an early stop codon marked by red asterisk. Residues boxed in red and yellow mark amino acid exchanges (indicated on top of alignment) in NcGln1 and NcGln2, respectively, of the *N*. *crassa* *gln-1b* mutant.

 So far, restoration of glutamine biosynthetic activity by heterologous GSI and GSII-encoding genes correlated with restoration of SM production probably due to restoration of wild type-like growth in submers culture under nitrogen-limiting conditions. Next we wanted to investigate if mutations in certain conserved amino residues would result in deregulation of NCR-sensitive genes (e.g. GA and bikaverin genes) without alteration of GS activity, or in the opposite phenotype, i.e. loss of GS activity but unchanged ability to produce SMs. To identify those residues, we performed an amino acid alignment between fungal, bacterial, plant and human GSI and GSII isozymes. The resulting phylogram shows a clear separation of the three classes of GS enzymes, and within the eukaryote clade, between fungi and animals on one hand, and photosynthetic eukaryotes on the other hand (Figure S2). In contrast to bacteria like *Streptomyces* spp. and *Rhizobium* spp. that posses GS proteins of various types, ascomycetous fungi harbor only GSII type proteins (Figure S2). Despite the separation into the three classes of GS, the amino acid alignment revealed a high degree of conservation between fungal, bacterial, plant and human GSI and GSII isozymes in 14 domains, probably responsible for substrate and ATP binding, enzymatic activity or the interaction among subunits (Figure 4) [4,5,80,81]. 

 To test our hypothesis, we generated mutants with specific amino acid substitutions in all 14 highly conserved domains (Figure 4; Table 2). Transformants carrying mutated gene copies under the control of the native *gln1* promoter were analyzed for their growth ability on solid minimal medium without glutamine and for biomass and SM formation in submers cultures under optimal GA-and bikaverin producing conditions (6 mM glutamine). In addition, the mutants were analyzed for the expression of NCR-sensitive and known NCR-insensitive GS target genes, such as *cipC* and *cpc* [47].

**Table 2:** Growth and secondary metabolism of the *F. fujikuroi* wild type, the ∆*gln1* mutant and mutants expressing point-mutated *gln1* gene copies.

**Strain**	**Growth on CD agar**	**DW in submers culture with 6 mM gln**	**DW in submers culture with 60 mM gln**	Production of Bikaverin*	Production of gibberellins*
WT	+	1.37	1.45	yes	yes
∆*gln1*	-	0.14	0.99	no	no
**D60/S62**	**-**	**0.86**	**1.23**	**yes**	**yes**
E131/E1331	-	0.15	1.32	no	no
E193	-	0.19	0.99	no	no
E200	+	1.27	1.49	yes	yes
E297	+	1.15	1.50	yes	yes
E330/R332	-	0.80	0.93	no	no
**G246/G248**	**-**	**0.97**	**1.63**	**yes**	**yes**
H250	-	0.16	1.33	no	no
H250/T255	-	0.18	1.34	no	no
L291	+	1.19	1.46	yes	yes
L76	+	1.02	1.64	yes	yes
R311	+	1.08	1.31	yes	yes
**S72/D73**	**-**	**0.93**	**1.54**	**yes**	**yes**
Y159	-	0.23	1.56	no	no

All strains were grown on CD agar and for three days in liquid minimal medium with 6 mM or 60 mM glutamine.

* gibberellins and bikaverin were determined only in nitrogen-limiting conditions (6 mM glutamine); CD- Czapek Dox medium; DW- dry weight (g/100 ml)

**Figure 5:**
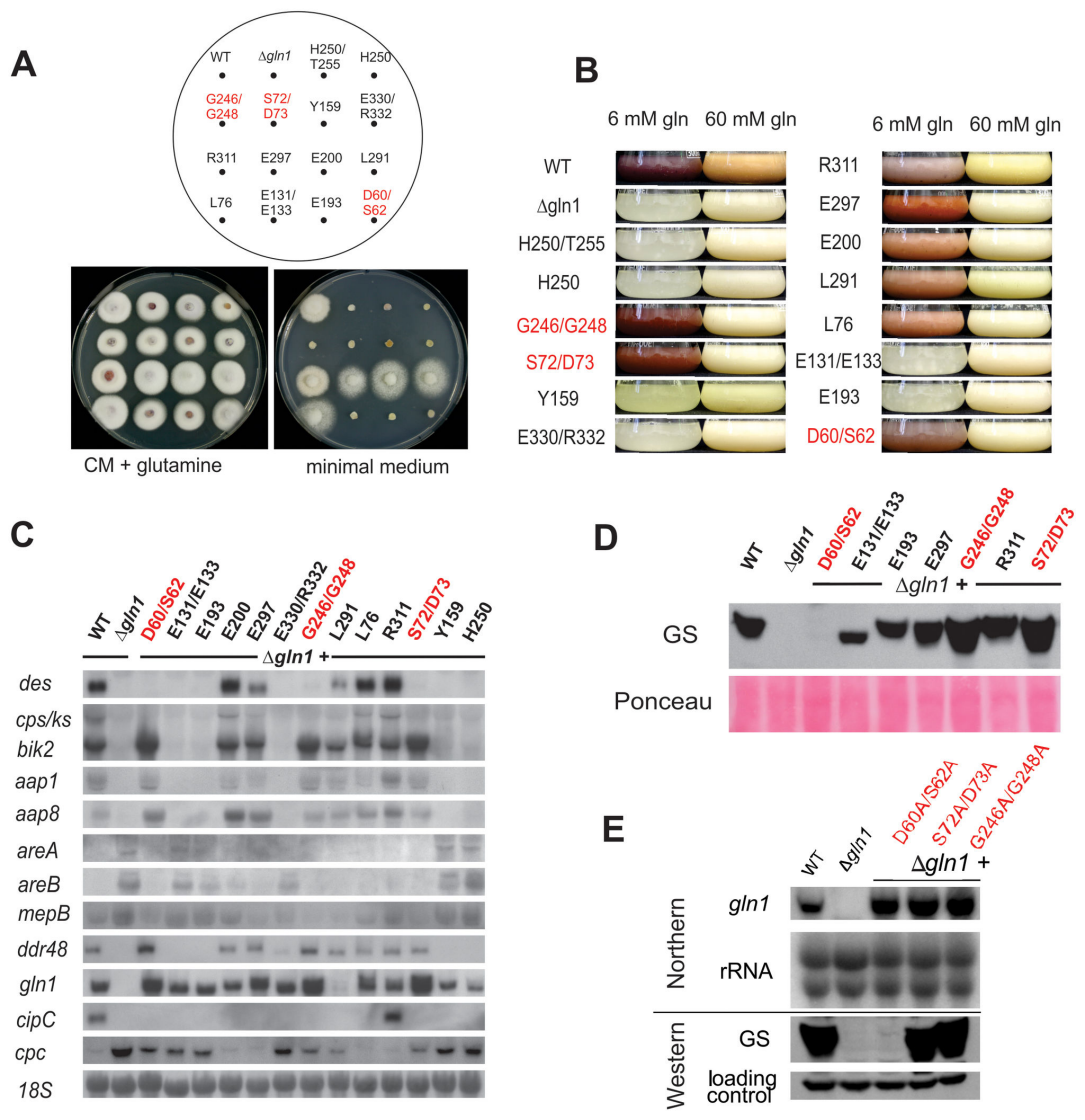
Characterization of site-directed Δ*gln1* complementation mutants. **A**. Plate assays comparing the growth of the wild type (WT), the Δ*gln1* mutant and different transformants complemented with a point-mutated copy of *gln1*. Complete medium (CM) contained 18 mM glutamine, while the minimal medium (CD) contained no additional nitrogen source. Pictures were taken after 3 days of cultivation. **B**. Pictures of the wild type (WT), the Δ*gln1* mutant and the point-mutated GS complementants (see Figure 4) grown for 3 days in ICI medium with either 6 mM (GA- and bikaverin-inducing (red coloration) conditions) or 60 mM glutamine as nitrogen source. **C**. Total RNA was isolated from mycelia grown for 5 days in ICI medium with 6 mM glutamine and used for northern blot analysis. *18S* rRNA was visualized as a loading control. Abbreviations: see legend of Figure 3.D. Total protein extract of the mycelia from **B** was used for western analysis using the polyclonal anti-TbGS antibodies and HRP-conjugated anti-rabbit secondary antibodies. Ponceau staining as loading control. **E**. Total RNA (Northern blot analysis) and protein (Western blot analysis) was isolated from mycelia of the wild type, the Δ*gln1* mutant and the transformants complemented with the three point-mutated copies of *gln1* which restore secondary metabolism but not glutamine formation. The strains were grown for 3 days in ICI medium with 6 mM glutamine.

 Of the 14 mutants generated, 5 (R311A, E297A, E200A, L291A, and L76A) were able to grow in a wild-type-like manner on agar plates without glutamine, indicating full enzymatic functionality, whereas 9 are glutamine auxotrophs showing no growth at all (Figure 5A). The 5 enzymatically functional mutants were able to restore *bik* and GA gene expression (Figure 5B) and concomitant bikaverin and GA formation (Figure 5B; Table 2) underlining the above mentioned correlation between GS activity and secondary metabolism. 

 Surprisingly, three of the nine glutamine auxotrophic mutants (D60A/S62A, G246A/G248A, S72A/D73A) highly expressed bikaverin biosynthetic genes (e.g. *bik3*) and exhibited wild-type-like bikaverin production (Figure 5B), while the signal for the GA biosynthetic gene *cps/ks* was almost non-detectable (Figure 5C). However, low concentrations of GA_3_ were measured by HPLC analysis in the three mutants in contrast to the Δ*gln1* mutant that does not produce any GAs (Table 2). Beside the restoration of secondary metabolism, these three mutants (D60A/S62A, G246A/G248A and S72A/D73A) also showed wild-type-like expression of the other NCR-sensitive genes (*aap1*, *app8*, *areA*, *areB* and *mepB*) and the GS target gene *ddr48*, despite the loss of glutamine-forming activity (Figure 5C). 

 In contrast, the expression pattern for the cross pathway gene *cpc* revealed a clear correlation with the ability to synthesize glutamine. Strains carrying non-functional *gln1* alleles (including D60A/S62A, G246A/G248A and S72A/D73A) displayed high, Δ*gln1*-like *cpc* expression (Figure 5C). As previously observed, the GS target gene *cipC* shows a specific expression pattern. Its expression seems to depend on a specific trait of either the *gln1* gene or its deduced protein independently of enzymatic functionality. Among the mutants with point-mutated *gln1* gene copies, only mutant R311A showed wild-type-like *cipC* expression (Figure 5C). The partial restoration of some wild-type phenotypes in the three regulatory mutants despite their lost catalytic activity is an indication for a complicated regulatory network centering on GS as one of the major players.

 To ensure that the mutated *gln1* gene copies are transcribed and translated into GS proteins in the different transformants, northern and western blot analyses were performed (Figure 5 D and E). In three independent experiments all mutants carrying a point-mutated gene copy demonstrated the expected strong expression (Figure 5E). To our surprise, no GS protein signal could be detected for the mutant D60A/S62A (Figure 5D and E). However, since this mutant displayed a wild-type-like phenotype regarding gene expression and secondary metabolism, the GS protein level in this mutant is probably below the detection sensitivity but sufficient for its regulatory function.

 In summary, the *gln1* site-directed mutagenesis approach clearly showed that it is possible to separate enzymatic activity and regulatory functions of the GS. Three of the fourteen GS mutants with specific point mutations have lost the ability to synthesize glutamine but were able to produce SMs and showed wild-type-like expression pattern for NCR-sensitive genes. These data strongly indicate that the *F. fujikuroi* GS is an important player in the nitrogen regulatory network. These regulatory functions are not dependent on its enzymatic activity and can now be pinpointed to specific amino acid residues.

### The three catalytic mutants have intermediate phenotypes between the Δ*gln1* mutant and the wild type

**Figure 6:**
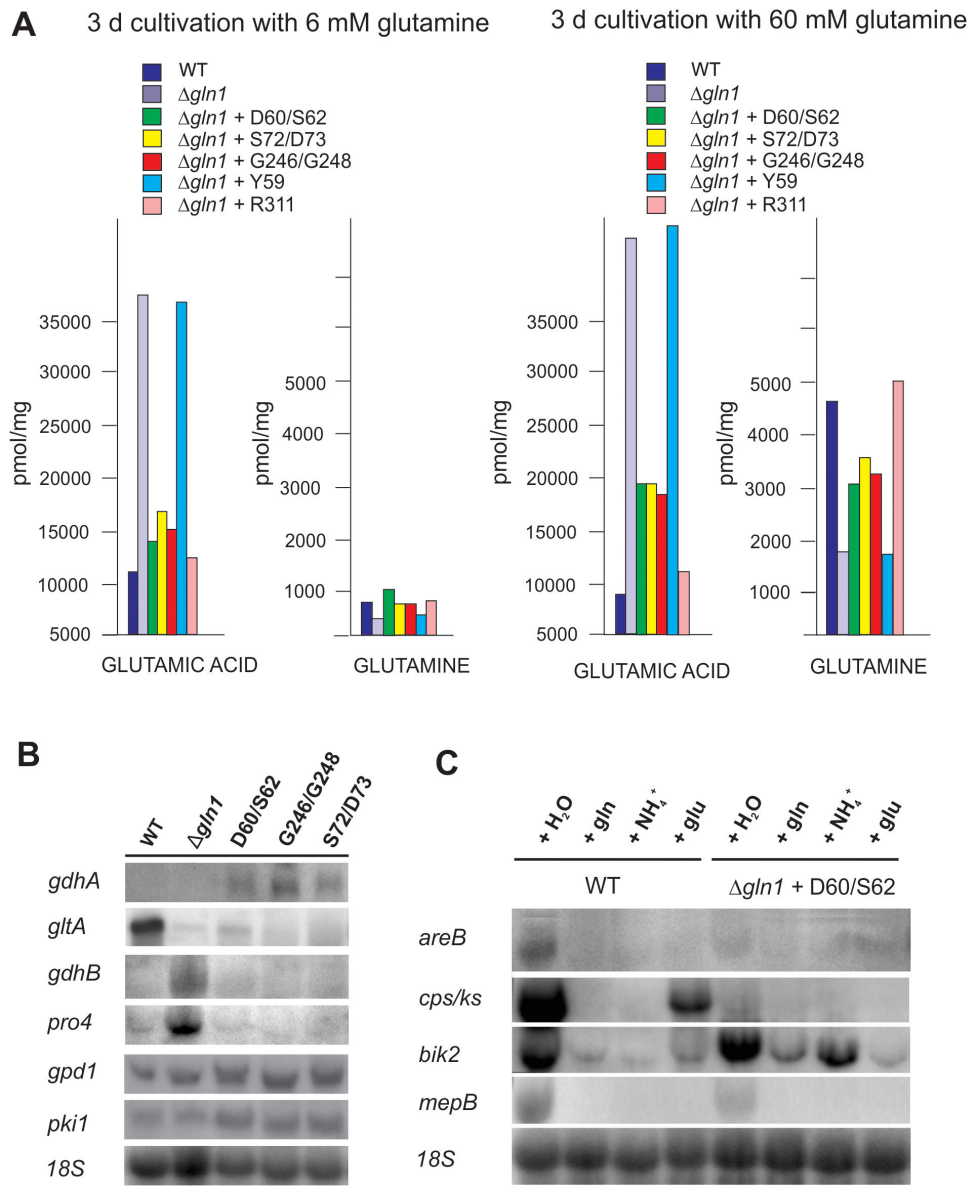
Impact of the *F. fujikuroi* GS on amino acid and carbon metabolism. **A**. Amino acid measurement of the wild-type, the Δ*gln1* mutant and three complemented strains carrying point-mutated *gln1* gene copies. The strains were grown for 3 days in ICI medium with 6 mM glutamine as nitrogen source. After 3 days the mycelia were harvested, lyophilized and used for amino acid analysis. **B**. Transcription of nitrogen and carbon metabolism genes. The wild type, the Δ*gln1* and the three deregulated mutants were grown for 3 days in ICI medium with 6 mM glutamine as N-source. The nitrogen-starved mycelia were harvested and the total RNA was used for northern analysis. *18S* rRNA was visualized as a loading control. Abbreviations: *gdhA*, NADH-dependent glutamate dehydrogenase; *gdhB*, NAD^+^-dependent glutamate dehydrogenase; *gltA*, glutamine oxoglutarate aminotransferase (GOGAT); *pro4*; glutamate-5-kinase; *gpd1*, glyceraldehyde-3-phosphate dehydrogenase gene; *pki1*, pyruvate kinase. **C**. The wild type and D60/S62 mutant strains were grown for 3 days in ICI medium containing 6 mM glutamine. Mycelia were harvested 30 minutes after addition of H_2_O, 60 mM glutamine (gln), 60 mM ammonium tartrate (NH_4_^+^) or potassium glutamate (glu) and hybridized with the indicated probes. *18S* rRNA was visualized as a loading control.

 To further characterize the three mutants with restored secondary metabolism (D60A/S62A, G246A/G248A, S72A/D73A), we compared their intracellular glutamine and glutamate concentrations to those of the wild type and the Δ*gln1* mutant. As controls we also analyzed the glutamine and glutamate pools in one mutant with a totally inactive GS variant (Y159) and one mutant with wild-type-like GS activity (R311). All strains were grown for three days in media with 6 mM and 60 mM glutamine, respectively. The most striking difference was the dramatically elevated glutamate level in the Δ*gln1* mutant and the completely inactive mutant Y159 in both media, probably due to the block of the dynamic shuttling between glutamate and glutamine by the glutamine synthetase/glutamate synthase (GS/GOGAT) cycle [82].Surprisingly, glutamate levels are significantly lower in the three catalytic mutants with restored secondary metabolism, displaying intermediate levels between the wild type and the Δ*gln1* mutant (Figure 6A). Glutamine levels are generally low in the wild type and all analyzed mutant strains after three days growth under nitrogen-limiting (6 mM glutamine) conditions, and significantly elevated in all strains in high nitrogen conditions. Interestingly, the three catalytic mutants (D60A/S62A, G246A/G248A and S72A/D73A) revealed again intermediate glutamine levels between the Δ*gln1* and Y59 mutants on the one hand, and the wild type and R311 mutant with an active GS, on the other hand (Figure 6A). 

 To explore what consequences different glutamate levels have on glutamate catabolism, we compared the expression of genes involved in the formation or degradation of glutamate between the wild type, the Δ*gln1* mutant and the three deregulated GS mutants (D60A/S62A, G246A/G248A and S72A/D73A) after three days growth in media with 6 mM glutamine (Figure 6B). The NADPH-dependent glutamate dehydrogenase encoding gene *gdhA* (glutamate producing) was only detectable in the three deregulated GS mutants (D60A/S62A, G246A/G248A and S72A/D73A), but neither in the Δ*gln1* mutant nor in the wild type. In contrast, the glutamate synthase (GOGAT) encoding gene, *gltA*, was weakly expressed in all tested strains compared to the wild type, probably due to glutamine starvation. However, the NAD^+^-dependent glutamate dehydrogenase (GdhB) encoding gene, *gdhB*, and the glutamate-5-kinase encoding gene, *pro4*, which is involved in proline metabolism, were up-regulated in the Δ*gln1* mutant and much lower expressed in the wild type and the three point mutants (Figure 6B). The up-regulation of *gdhB* and *pro4* in the Δ*gln1* mutant is not surprising. Both encoded enzymes use glutamate as substrate, and the high expression of these two genes might be a response to the accumulation of glutamate in the Δ*gln1* strain. These data indicate that the three regulatory mutants show an intermediate phenotype between the wild type and the Δ*gln1* mutant regarding expression of genes involved in glutamate metabolism. 

 One of the most obvious phenotypes of the Δ*gln1* mutant is the significantly reduced growth in media with high amounts (440 mM) of glucose and 6 mM glutamine compared to the wild type under the same conditions (Table 2). These data indicate that the mutant is not able to utilize glucose as efficiently as the wild type, as indicated by the reduced dry weight formation (only 15% dry weight compared to the wild type). Comparable mutants such as the ΔΔ*gdhA*/*gltA* double and the Δ*aar1* single deletion mutants with strict glutamate and lysine auxotrophy, respectively, show neither growth defects on media with the complementing amino acid substrates nor impairments in secondary metabolism [48,83]. Therefore, we suggested additional consequences of the Δ*gln1* deletion on primary metabolism, e.g. on the ability to efficiently utilize glucose and generate ATP. To prove this hypothesis, we compared the growth of the wild type, the Δ*gln1* and the mutants with point-mutated *gln1* gene copies in the standard minimal medium with 6 mM glutamine and 440 mM glucose. All prototrophic mutants grow similarly as the wild type while the glutamine auxotrophic strains accumulate significantly less biomass similar to the Δ*gln1* mutant. Surprisingly, the three deregulated mutants (D60A/S62A, G246A/G248A and S72A/D73A) produced five to six-fold more dry weigth than the Δ*gln1* mutant and the other auxotrophic strains, thus demonstrating an intermediate growth between the wild type and the Δ*gln1* mutant (Table 2). 

 To show if the inability to efficiently utilize glucose might be the reason for loss of secondary metabolism in the glutamine auxotrophic mutants, we compared the growth of the wild type, the Δ*gln1* and the three specific mutants in nitrogen-limited media (6 mM glutamine) with or without glucose (glutamine serves as sole nitrogen and carbon source). While all strains grew poorly without glucose, the auxotrophic strains accumulated even more biomass than the wild type under these conditions (Table S3). Neither the wild type nor the Δ*gln1* or the three site-directed mutants were able to promote GA nor bikaverin production without glucose indicating that secondary metabolism depends on optimal energy balance and carbon source availibility. 

 In summary, the GS seems to play an important role also in carbon/energy metabolism. Most of the site-mutated glutamine auxotrophic mutants are affected in their ability to efficiently utilize glucose, to grow in a wild-type-like manner and to produce SMs. However, only those three auxotrophic mutants which show almost wild-type-like growth in the standard medium with glucose and 6 mM glutamine were also able to produce SMs suggesting that the GS is essential for an effective and balanced carbon/energy metabolism.

### The GS most likely senses NH_4_^+^ availibility

 The mutant D60A/S62A contains a mutation in a conserved domain probably involved in NH_4_^+^ binding. To examine if this mutation leads to altered expression pattern of NCR sensitive genes after addition of ammonium to nitrogen-starving mycelia, we grew this mutant and the wild type under glutamine limiting conditions for three days before adding either glutamine, NH_4_^+^, glutamate or no nitrogen source (starvation). Gene expression of NCR sensitive primary (*mepB*) and SM biosynthetic (*cps/ks*; *bik2*) genes was monitored 30 minutes after addition of the respective nitrogen sources. In the wild type, glutamine, NH_4_^+^ and to a lesser extent glutamate repressed expression of the AreA-dependent (*cps/ks, mepB*) and -independent (*bik2*) genes (Figure 6C). In the D60A/S62A mutant carrying a mutation in the putative NH_4_^+^ binding site, addition of glutamine and glutamate but not NH_4_^+^ caused a decrease in *bik2* expression compared to the water control (Figure 6C). These data indicate that NH_4_^+^ does not act as repressing nitrogen source in this mutant and that the mutations in the putative NH_4_^+^ binding site D60/S62 cause loss of NH_4_^+^ sensing and subsequent loss of wiring the repressing signal towards bikaverin gene expression.

 Summarizing, the data provided in this study strongly support our hypothesis that the GS plays an important role in the nitrogen regulation network. The GS is involved in nitrogen sensing, regulation of NCR-sensitive genes as well as primary, secondary and carbon/energy metabolism. Using a site directed mutagenesis approach we were able to demonstrate that the enzymatic function of the GS can partially be separated from the additional regulatory and sensory functions. The inability of the Δ*gln1* mutant to produce SMs is probably due to its failure to utilize glucose for sufficient ATP formation. Furthermore, the putative NH_4_^+^ binding site D60/S62 in the GS seems to be responsible for NH_4_^+^ sensing independently of the enzymatic functionality. 

## Discussion

**Figure 7:**
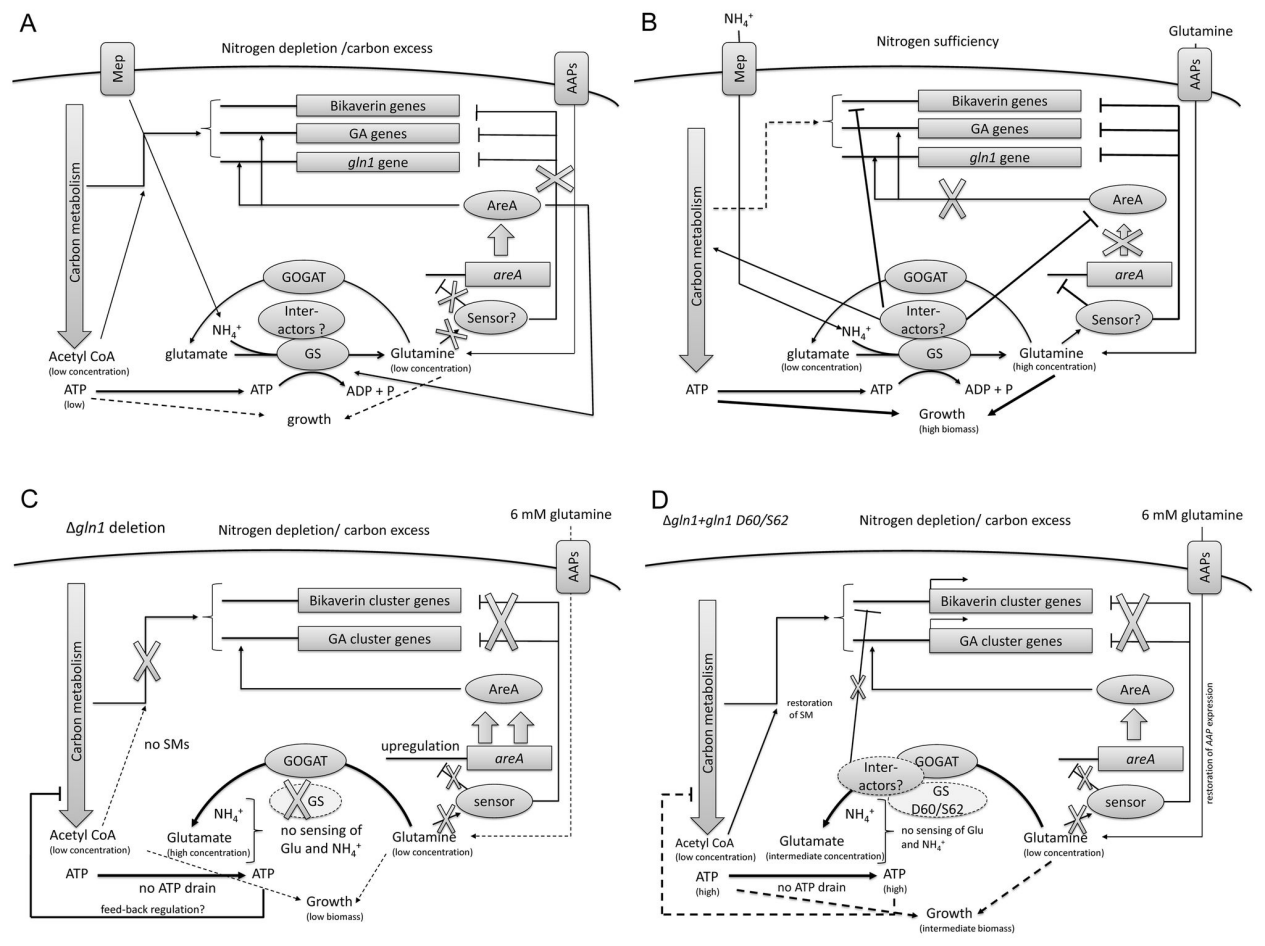
Proposed model of GS-dependent regulation in *F*. ***fujikuroi***. **A**. Growth of the wild type under nitrogen-limiting conditions. GS transcript and protein levels are under positive control of AreA, similar to the gibberellic acid (GA) cluster genes. Bikaverin genes are also highly expressed in an AreA-independent manner probably due to the low glutamine pool in the cell which is sensed by a putative intracellular sensor. **B**. Growth of the wild type under nitrogen-sufficient conditions. Excess of glutamine is detected by the postulated GS-independent sensor protein, exerting inhibition of AreA (no GA gene expression) and bikaverin cluster gene expression. Ammonium and glutamate are sensed by the GS itself. This signal is transduced by one or multiple putative GS interactor proteins that enhance carbon metabolism and inhibit expression nitrogen metabolite repression-sensitive gene expression. The inducing or repressing effect of these interactors on SM gene expression depends on the nitrogen status of the cell. Beside the direct regulatory impact of the GS by binding interactor(s), its ATP consuming activity depletes the intracellular ATP pool, which in turn prohibits the ATP-mediated inhibition of the carbon metabolism. An efficient carbon metabolism is required for the expression of SM cluster genes. To maintain the ATP-consuming GS activity the produced glutamine is reverted to glutamate by the activity of the glutamine oxoglutarate aminotransferase (GOGAT). **C**. Situation in the Δ*gln1* mutant. No sensing of ammonium and glutamate, and no interaction with putative interactors are possible. The missing ATP drain probably results in negative feedback regulation of carbon metabolism leading to significantly reduced growth and down-regulation of secondary metabolism despite the elevated *areA* expression. The low glutamine concentration can not overcome the the disturbed glucose consumption. **D**. In the D60/S62 mutant, ammonium and glutamate are probably not sensed. The presence of a GS protein (though mutated and unable to produce glutamine) allows the binding to yet unknown interactors thereby partially restoring secondary metabolism, carbon metabolism and growth in low glutamine/high glucose conditions. Abbreviations: Mep- ammonium permease; AAP- amino acid permease.

 In this work, we performed detailed expression analyses and SM profiling of the wild type, the Δ*gln1* mutant and transformants carrying heterologous or specifically mutated GS-encoding alleles in order to show that the GS is involved in nitrogen sensing, regulation of NCR-sensitive primary and secondary metabolism genes and carbon/energy metabolism. Based on this new information, we are summarizing all aspects of our study in a model for the GS-dependent regulation network 1) in the wild type under nitrogen-limiting and sufficient conditions (Figure 7A, B), and 2) in the Δ*gln1* and D60A/S62A mutants (Figure 7C, D). 

### The GS is involved in nitrogen sensing and regulation of *areA* expression

 Previously we have shown that GA and bikaverin gene expression is repressed by nitrogen in an AreA-dependent and independent manner, respectively, and that AreA also regulates the expression of *gln1* [44,47] (Figure 7A, B). Here we demonstrate for the first time that deletion of *areA* also resulted in significant down-regulation of GS protein levels. In addition, we provide evidence that the expression levels of the regulatory genes *areA*, *areB* and *nmr* depend on the GS: the genes are strongly upregulated in the *gln1* deletion mutant compared to the wild type despite the addition of sufficient amounts of different nitrogen sources (Figure 2A; Figure 7B, C). These data suggest that the mutant is not able to sense these high nitrogen concentrations as fast as the wild type. The up-regulation of *areA*, *areB* and *nmr* is probably an indirect effect of the even more reduced intracellular glutamine pool in the deletion mutant which is sensed by a yet unknown intracellular glutamine sensor (Figure 7C, shown for *areA*). High amounts of the two GS substrates, glutamate and NH_4_^+^ allowed a persistent expression of *areA* and *areB* (not included in scheme) in the deletion mutant, while glutamine and NO_3_^-^ (not included in scheme) seem to be sensed by a GS-independent mechanism, resulting in down-regulation of the regulatory genes (Figure 2B; Figure 7C). These data are in accordance with findings in *N. crassa* or *F. fujikuroi*, where inhibition of GS by MSX or mutations in the GS-encoding genes resulted in relieved gene repression by NH_4_^+^ and glutamate but not by glutamine [73,84,85].

### The GS is involved in regulation of primary and secondary metabolism

 The most unexpected phenotypes of the Δ*gln1* mutant were the loss of SMs (GA and bikaverin), significantly reduced growth with low glutamine and altered expression of specific sets of genes upon varying nitrogen conditions compared to the wild type. Based on the high conservation of GS proteins, we expressed several GS-encoding genes of prokaryotic (*Streptomyces*) and eukaryotic (*N. crassa*) origin in the *F. fujikuroi* mutant. We expected a restoration of glutamine prototrophy as shown for the *A. nidulans* Δ*glnA* mutant complemented with the *glnA* gene of the cyanobacterium *Anabaena* sp. [86]. However, we did not expect restoration of fungus-specific secondary metabolism by any heterologous GS. To our surprise, both *N. crassa* genes, *Ncgln1* and *Ncgln2*, restored growth and secondary metabolism indicating that each of the *N. crassa* GS proteins alone can assemble to fully functional GS multimers. These data fit to the prototrophic phenotypes of single *Ncgln1* and *Ncgln2* deletion mutants, respectively (Figure S1), and are in accordance with prototrophic single deletion mutants in *Streptomyces* spp. [87]. Even more surprising was the full restoration of both glutamine prototrophy and the ability to produce GAs and bikaverin by the GSI-type *gln1* and the GSII-type *glnII* of *S. coelicolor*, although the genes share only 22.2 % and 48.3 % of amino acid identity with the GS of *F. fujikuroi*, respectively. Since it is very unlikely that bacterial GS proteins can control the activity of specific transcription factors in the background of an eukaryotic cell, we suggest that the restoration of SM production is due to the restored formation of glutamine and subsequent wild-type-like growth of these mutants. 

 A specific indicator for full GS functionality is the expression of the recently identified GS-dependent gene *cipC* [47]. Its expression can not be restored by feeding the mutant with glutamine but depends on the presence of a highly similar GS protein such as NcGln2. *CipC* expression is not restored in mutants carrying prototrophic *gln1* and *glnII* genes from *S. coelicolor* or *Ncgln1* from *N. crassa*. These data indicate that the *F. fujikuroi* GS fulfills several regulatory functions probably by interacting with yet unknown and highly specific regulatory proteins. Strikingly, beside the *Ncgln2* wild-type gene, the defective *Ncgln2* gene products from both the *gln-1a* and *gln-1b* mutants also showed expression of *cipC* supporting our assumption that specific GS-interacting proteins could exist (Figure 7). 

 As the heterologous expression of *N. crassa* and *S. coelicolor* GS-encoding wild type genes resulted in concomitant restoration of almost all wild-type phenotypes, we performed a site directed mutagenesis approach by specifically mutating all 14 highly conserved amino acid motifs in order to separate the different functions of the GS. Similar studies of GS have been reported for *Bacillus subtilis* [88], but not for any eukaryotic GS protein so far.

 Three of the fourteen generated mutants (D60A/S62A, G246A/G248A and S72A/D73A) expressing specific point-mutated *F. fujikuroi gln1* gene alleles are strictly glutamine auxotrophic but retained some but not all regulatory effects of wild-type GS. Thus, all three mutants are able to produce wild-type levels of bikaverin and low amounts of GAs. It is likely that the expression of bikaverin biosynthetic genes depends mainly on a functional carbon and energy (ATP) metabolism (as discussed below) and the absence of AreA-independent nitrogen repression, while the GA gene expression needs active AreA and AreB regulators in addition (Figure 7A). This differing regulatory function of the GS on the expression of the two distinct SMs is most likely due to the different mechanisms of nitrogen repression for GAs and bikaverin and the involvement of different yet unknown regulatory protein(s). These regulatory proteins might interact with the GS under distinct circumstances, allowing an induction of the carbon metabolism and simultaneous repression of *areA* (Figure 7). 

 In contrast to the wild type, one of the generated GS mutants, D60A/S62A, having point mutations in a putative NH_4_^+^-binding site, revealed derepressed *bik2* levels when exposed to ammonium. This result argues for the importance of these residues to sense and transduce this specific repressing signal via a putative interaction partner (Figure 7B). 

 In addition to partial restoration of secondary metabolism, the three mutants show a wild-type-like expression pattern for a set of NCR sensitive genes, but Δ*gln1* mutant-like behavior for another set of genes, such as the cross pathway regulatory gene *cpc* and genes involved in glutamate metabolism. 

### The GS is tightly linked to energy metabolism

 One of the most surprising phenotypes of the ∆*gln1* mutant is the drastically impaired growth under standard GA-and bikaverin-inducing conditions (6 mM glutamine) compared to the wild type. While the wild type accumulates about 1.4 g/l dry weight, the mutant produces only about 0.2 g/l dry weight under identical nitrogen conditions (Table 2). Northern blot analyses and amino acid concentration measurements suggest that the GS has a strong impact on anabolic amino acid pathways in *F. fujikuroi*. The ∆*gln1* mutant seems to be unable to maintain metabolic homeostasis due to the interrupted GS-GOGAT cycle, the connecting step between carbon and nitrogen metabolism. As a consequence, the isocitrate lyase-encoding gene (*ICL*), the key enzyme of glyoxylate cycle [47], and several other genes involved in glutamate metabolism, are miss-regulated (Figure 6B). However, the altered amino acid (glutamate, glutamine) concentrations in the Δ*gln1* mutant compared to the wild type seem unlikely to cause the severe growth restrictions, since the deletion mutant displayed enhanced growth when glutamine was supplied as sole carbon and nitrogen source (Table 2). From these data we conclude that the Δ*gln1* mutant is defective in glucose consumption and subsequently in providing sufficient ATP to allow wild-type-like growth. Our assumption is in accordance with earlier findings in *N. crassa* where inhibition of GS-activity resulted in strong inhibition of carbon catabolism and growth [17,89]. Most likely, the reason for this dependency of carbon catabolism on the GS activity is the high ATP consumption by the functional GS, creating an ATP drain, which in turn activates the catabolism of carbon sources [17,89]. This suggestion is supported by the finding that a *N. crassa gdhA* deletion mutant, inhibited in GS activity, was able to grow with glutamine as sole carbon- and nitrogen source in contrast to the wild type [90]. The growth defect of the wild type under these conditions was attributed to the highly active ATP-consuming glutamine/glutamate cycling (GS-GOGAT) [91] contributing to an ATP-drain that would drive the ATP-producing glycolysis in the presence of glucose [90,92]. Similar observations have been reported in *S. cervisiae* [93]. The data obtained in this work make a comparable mechanism in *F. fujikuroi* highly conceivable. A disrupted ATP-consuming glutamate/glutamine cycling mechanism could explain the low growth rate of the *∆gln1* mutant with low glutamine concentrations when glucose is present. Glutamate accumulates due to the missing conversion back to glutamine by the GS, and the unused ATP would accumulate and repress efficient glycolysis (Figure 7C). Evidence that an inactivated carbon/energy metabolism in the Δ*gln1* mutant is responsible for the loss of SMs comes from the fact that the wild type did not produce bikaverin or GAs when glucose was absent and glutamine served as the only nitrogen and carbon source. In accordance with this hypothesis, the three deregulated mutants showed a partial restoration of the growth defect. Although they are strictly glutamine auxotrophic, they produce significantly more biomass than the *∆gln1* mutant indicating that the enzymatically inactive GS enzymes with mutations in D60/S62, G246/G248 and S72/D73 residues are partially able to regulate the energy metabolism (Figure 7D). 

 In summary, detailed comparisons of growth rates, gene expression analyses and SM production between the wild type, the Δ*gln1* mutant and transformants carrying heterologous or specifically mutated GS-encoding alleles allowed us to postulate that the GS not only provides glutamine for cell viability, but also functions as a regulatory hub by controlling gene expression. We showed that the GS most likely senses the abundance of its substrates, NH_4_^+^ and glutamate, and transmits this information to downstream targets by an as yet unidentified mechanism that involves AreA (GA biosynthesis) and other yet unknown (bikaverin biosynthesis) regulators. Our data indicate that the GS is tightly linked to carbon/energy metabolism, thereby connecting primary to secondary metabolism (Figure 7).

 Furthermore, by generating specific site-directed GS mutants we were able to separate the enzymatic function from the role as a major regulator of many metabolic processes. Three point mutations led to partial restoration of secondary metabolism, wild-type-like growth behavior, intracellular amino acid concentrations, and gene expression patterns despite their inability to produce glutamine. Furthermore, the putative NH_4_^+^ binding site D60/S62 in the GS seems to be responsible for NH_4_^+^ sensing independently of enzymatic functionality. These data support our assumption of a regulatory role of the mere GS protein disclosing the possibility for putative direct interaction partners. We therefore suggest that the *F. fujikuroi* GS, similar to bacterial GS, belongs to the group of bifunctional “trigger enzymes” that recognize their substrates and often undergo structural alterations upon this interaction. These enzymes are active in metabolism and in the same time are able to regulate gene expression, probably by their interference with transcriptional regulators [94]. Unraveling the effects caused by an altered metabolic status (carbon and energy) in the Δ*gln1* mutant and/or by altered signal transduction of putative GS interaction partners will be a fascinating future task.

## Supporting Information

Figure S1 **Gln1 and Gln2 have redundant functions in *N. crassa*.** A. Growth of the *N. crassa* wild type (WT) and the Δ*gln1* and Δ*gln2* single mutants on minimal medium (MM) and MM supplemented with glutamine. Maximum linear hyphal extension was determined on race tubes. Error bars indicate standard deviations calculated from three independent experiments. **B**. Growth of the heterokaryotic double mutant after three days of inoculation compared to the wild type and the single mutants on selective medium (200 μg/ml Nourseothricin) with (top) and without glutamine (bottom). The wild type and the single mutants carried the same resistance marker (Nat^R^), which was used to construct the double mutant.
(TIF)

Figure S2 **Phylogram of prokaryotic and eukaryotic GS proteins.** Protein sequences for the phylogram were retrieved from the Protein Knowledgebase (UniProtKB) by searching for reviewed sequences of glutamine synthetases. Proteins are assigned by Swiss-Prot identifier (sp_). The *N. crassa* NcGln2 sequence (NCU_06724.5) was retrieved from the Broad Institute Database. Relevant sequences for this study are indicated in bold, the *F. fujikuroi* sequence in red.
(DOCX)

Table S1 **Oligonucleotides used in this study.**
(DOCX)

Table S2 **Identified mutations in the GS-encoding genes *Ncgln1* (NCU04856) and *Ncgln2* (NCU06724) of mutant strains gln-1a and gln-1b.**
(DOCX)

Table S3 **Growth and secondary metabolite production of the *F. fujikuroi* wild type, the Δ*gln1* mutant and three complementants carrying site-directed mutations of conserved residues of the *F. fujikuroi* GS.** All strains were grown in minimal medium (MM) with 6 mM or 60 mM glutamine with 440 mM glucose (+C) or without glucose (-C).
(DOCX)

## References

[1] Wedler FC, Horn BR (1976) Catalytic mechanisms of glutamine synthetase enzymes. Studies with analogs of possible intermediates and transition states. J Biol Chem 251: 7530-7538. PubMed: 12170. 12170

[2] Liaw SH, Eisenberg D (1994) Structural model for the reaction mechanism of glutamine synthetase, based on five crystal structures of enzyme-substrate complexes. Biochemistry 33: 675-681. doi:10.1021/bi00169a007. PubMed: 7904828. 7904828

[3] Van Rooyen JM, Abratt VR, Belrhali H, Sewell T (2011) Crystal structure of type III glutamine synthetase: Surprising reversal of the inter-ring interface. Structure 19: 471-483. doi:10.1016/j.str.2011.02.001. PubMed: 21481771. 21481771

[4] Unno H, Uchida T, Sugawara H, Kurisu G, Sugiyama T et al. (2006) Atomic structure of plant glutamine synthetase. J Biol Chem 281: 29287-29296. doi:10.1074/jbc.M601497200. PubMed: 16829528. 16829528

[5] Krajewski WW, Collins R, Holmberg-Schiavone L, Jones TA, Karlberg T et al. (2008) Crystal structures of mammalian glutamine synthetases illustrate substrate-induced conformational changes and provide opportunities for drug and herbicide design. J Mol Biol 375: 217-228. doi:10.1016/j.jmb.2007.10.029. PubMed: 18005987. 18005987

[6] He YX, Gui L, Liu YZ, Du Y, Zhou Y (2009) Crystal structure of *Saccharomyces* *cerevisiae* glutamine synthetase Gln1 suggests a nanotube-like supramolecular assembly. Proteins 76: 249-254. doi:10.1002/prot.22403. PubMed: 19322816. 19322816

[7] Eichinger L, Pachebat JA, Glöckner G, Rajandream MA, Sucgang R et al. (2005) The genome of the social amoeba *Dictyostelium* *discoideum* . Nature 435: 43-57. doi:10.1038/nature03481. PubMed: 15875012. 15875012 PMC1352341

[8] Kinoshita S, Isu S, Kaneko G, Yamada H, Hara T et al. (2009) The occurrence of eukaryotic type III glutamine synthetase in the marine diatom *Chaetoceros* *compressum* . Mar Genomics 2: 103-111. doi:10.1016/j.margen.2009.06.003. PubMed: 21798178. 21798178

[9] Kumada Y, Takano E, Nagaoka K, Thompson CJ (1990) *Streptomyces* *hygroscopicus* has two glutamine synthetase genes. J Bacteriol 172: 5343-5351. PubMed: 1975585. 1975585 10.1128/jb.172.9.5343-5351.1990 PMC213198

[10] Mathis R, Gamas P, Meyer Y, Cullimore JV (2000) The presence of GSI-like genes in higher plants: Support for the paralogous evolution of GSI and GSII genes. J Mol Evol 50: 116-122. PubMed: 10684345. 10684345 10.1007/s002399910013

[11] Robertson DL, Tartar A (2006) Evolution of glutamine synthetase in heterokonts: Evidence for endosymbiotic gene transfer and the early evolution of photosynthesis. Mol Biol Evol 23: 1048-1055. doi:10.1093/molbev/msj110. PubMed: 16495348. 16495348

[12] Robertson DL, Smith GJ, Alberte RS (2001) Glutamine synthetase in marine algae: New surprises from an old enzyme. J Phycol 37: 793-795. doi:10.1046/j.1529-8817.2001.01057.x.

[13] Wyatt K, White HE, Wang L, Bateman OA, Slingsby C (2006) Lengsin is a survivor of an ancient family of class I glutamine synthetases re-engineered by evolution for a role in the vertebrate lens. Structure 14: 1823-1834. doi:10.1016/j.str.2006.10.008. PubMed: 17161372. 17161372 PMC1868402

[14] Reuther J, Wohlleben W (2007) Nitrogen metabolism in *Streptomyces* *coelicolor*: Transcriptional and post-translational regulation. J Mol Microbiol Biotechnol 12: 139-146. doi:10.1159/000096469. PubMed: 17183221. 17183221

[15] Dávila G, Lara M, Guzmán J, Mora J (1980) Relation between structure and function of glutamine synthetase. Biochem Biophys Res Commun 92: 134-140. doi:10.1016/0006-291X(80)91530-2. PubMed: 6101946. 6101946

[16] Lara M, Blanco L, Campomanes M, Calva E, Palacios R et al. (1982) Physiology of ammonium assimilation in *Neurospora* *crassa* . J Bacteriol 150: 105-112. PubMed: 6120927. 6120927 10.1128/jb.150.1.105-112.1982 PMC220087

[17] Mora J (1990) Glutamine metabolism and cycling in *Neurospora* *crassa* . Microbiol Rev 54: 293-304. PubMed: 2145504. 2145504 10.1128/mr.54.3.293-304.1990 PMC372778

[18] Galagan JE, Calvo SE, Borkovich KA, Selker EU, Read ND et al. (2003) The genome sequence of the filamentous fungus *Neurospora* *crassa* . Nature 422: 859-868. doi:10.1038/nature01554. PubMed: 12712197. 12712197

[19] Caddick MX, Peters D, Platt A (1994) Nitrogen regulation in fungi. Antonie Van Leeuwenhoek 65: 169-177. doi:10.1007/BF00871943. PubMed: 7847882. 7847882

[20] Dunn-Coleman NS, Garrett RH (1980) The role of glutamine synthetase and glutamine metabolism in nitrogen metabolite repression, a regulatory phenomenon in the lower eukaryote *Neurospora* *crassa* . Mol Gen Genet 179: 25-32. doi:10.1007/BF00268442. PubMed: 6109228. 6109228

[21] Dunn-Coleman NS, Tomsett AB, Garrett RH (1981) The regulation of nitrate assimilation in *Neurospora* *crassa*: Biochemical analysis of the *nmr-1* mutants. Mol Gen Genet 182: 234-239. doi:10.1007/BF00269663. PubMed: 6457234. 6457234

[22] Platt A, Langdon T, Arst HN, Kirk D, Tollervey D et al. (1996) Nitrogen metabolite signalling involves the C-terminus and the GATA domain of the *Aspergillus* transcription factor AREA and the 3' untranslated region of its mRNA. EMBO J 15: 2791-2801. PubMed: 8654376. 8654376 PMC450215

[23] Montanini B, Betti M, Márquez AJ, Balestrini R, Bonfante P et al. (2003) Distinctive properties and expression profiles of glutamine synthetase from a plant symbiotic fungus. Biochem J 373: 357-368. doi:10.1042/BJ20030152. PubMed: 12683951. 12683951 PMC1223491

[24] Magasanik B, Kaiser CA (2002) Nitrogen regulation in *Saccharomyces* *cerevisiae* . Gene 290: 1-18. doi:10.1016/S0378-1119(02)00558-9. PubMed: 12062797. 12062797

[25] Wiame JM, Grenson M, Arst HN (1985) Nitrogen catabolite repression in yeasts and filamentous fungi. Adv Microb Physiol 26: 1-88. doi:10.1016/S0065-2911(08)60394-X. PubMed: 2869649. 2869649

[26] Wiemann P, Tudzynski B (2013) The nitrogen regulation network and its impact on secondary metabolism and pathogenicity. In: Brown DW Proctor RH *Fusarium*: Genomics, Molecular and Cellular Biology. Norwich, UK: Caister Academic Press p. 7.

[27] Camargo A, Llamas A, Schnell RA, Higuera JJ, González-Ballester D et al. (2007) Nitrate signaling by the regulatory gene NIT2 in *Chlamydomonas* . Plant Cell 19: 3491-3503. doi:10.1105/tpc.106.045922. PubMed: 18024571. 18024571 PMC2174885

[28] Rutherford JC, Chua G, Hughes T, Cardenas ME, Heitman J (2008) A Mep2-dependent transcriptional profile links permease function to gene expression during pseudohyphal growth in *Saccharomyces* *cerevisiae* . Mol Cell Biol 19: 3028-3039. doi:10.1091/mbc.E08-01-0033. PMC2441671 18434596

[29] Crespo JL, Powers T, Fowler B, Hall MN (2002) The TOR-controlled transcription activators GLN3, RTG1, and RTG3 are regulated in response to intracellular levels of glutamine. Proc Natl Acad Sci U_S_A 99: 6784-6789. doi:10.1073/pnas.102687599. PubMed: 11997479. 11997479 PMC124480

[30] Georis I, Tate JJ, Cooper TG, Dubois E (2011) Nitrogen-responsive regulation of GATA protein family activators Gln3 and Gat1 occurs by two distinct pathways, one inhibited by rapamycin and the other by methionine sulfoximine. J Biol Chem 286: 44897-44912. doi:10.1074/jbc.M111.290577. PubMed: 22039046. 22039046 PMC3248002

[31] Gunka K, Commichau FM (2001) Control of glutamate homeostasis in *Bacillus* *subtilis*: a complex interplay between ammonium assimilation, glutamate biosynthesis and degradation. Mol Microbiol 85: 213-224. PubMed: 22625175. 10.1111/j.1365-2958.2012.08105.x 22625175

[32] Wray LV, Zalieckas JM, Fisher SH (2001) *Bacillus* *subtilis* glutamine synthetase controls gene expression through a protein-protein interaction with transcription factor TnrA. Cell 107: 427-435. doi:10.1016/S0092-8674(01)00572-4. PubMed: 11719184. 11719184

[33] Liaw SH, Jun G, Eisenberg D (1994) Interactions of nucleotides with fully unadenylylated glutamine synthetase from *Salmonella* *typhimurium* . Biochemistry 33: 11184-11188. doi:10.1021/bi00203a014. PubMed: 7727369. 7727369

[34] Bömke C, Tudzynski B (2009) Diversity, regulation, and evolution of the gibberellin biosynthetic pathway in fungi compared to plants and bacteria. Phytochemistry 70: 1876-1893. doi:10.1016/j.phytochem.2009.05.020. PubMed: 19560174. 19560174

[35] Wiemann P, Willmann A, Straeten M, Kleigrewe K, Beyer M et al. (2009) Biosynthesis of the red pigment bikaverin in *Fusarium* *fujikuroi*: Genes, their function and regulation. Mol Microbiol 72: 931-946. doi:10.1111/j.1365-2958.2009.06695.x. PubMed: 19400779. 19400779

[36] Studt L, Wiemann P, Kleigrewe K, Humpf HU, Tudzynski B (2012) Biosynthesis of fusarubins accounts for pigmentation of *Fusarium* *fujikuroi* perithecia. Appl Environ Microbiol 78: 4468-4480. doi:10.1128/AEM.00823-12. PubMed: 22492438. 22492438 PMC3370568

[37] Studt L, Humpf HU, Tudzynski B (2013) Signaling governed by G proteins and cAMP is crucial for growth, secondary metabolism and sexual development in *Fusarium* *fujikuroi* . PLOS ONE 8: e58185. doi:10.1371/journal.pone.0058185. PubMed: 23469152. 23469152 PMC3585259

[38] Studt L, Troncoso C, Gong F, Hedden P, Toomajian C et al. (2012) Segregation of secondary metabolite biosynthesis in hybrids of *Fusarium* *fujikuroi* and *Fusarium* *proliferatum* . Fungal Genet Biol 49: 567-577. doi:10.1016/j.fgb.2012.05.005. PubMed: 22626844. 22626844

[39] Kleigrewe K, Niehaus EM, Wiemann P, Tudzynski B, Humpf HU (2012) New approach via gene knockout and single-step chemical reaction for the synthesis of isotopically labeled fusarin C as an internal standard for the analysis of this *Fusarium* mycotoxin in food and feed samples. J Agric Food Chem 60: 8350-8355. doi:10.1021/jf302534x. PubMed: 22877497. 22877497

[40] Díaz-Sánchez V, Avalos J, Limón MC (2012) Identification and regulation of *fusA*, The polyketide synthase gene responsible for fusarin production in *Fusarium* *fujikuroi* . Appl Environ Microbiol 78: 7258-7266. doi:10.1128/AEM.01552-12. PubMed: 22865073. 22865073 PMC3457117

[41] Brown DW, Butchko RAE, Busman M, Proctor RH (2012) Identification of gene clusters associated with fusaric acid, fusarin, and perithecial pigment production in *Fusarium* *verticillioides* . Fungal Genet Biol 49: 521-532. doi:10.1016/j.fgb.2012.05.010. PubMed: 22652150. 22652150

[42] Wiemann P, Sieber CMK, von Bargen KW, Studt L, Niehaus EM et al. (2013) Deciphering the cryptic genome: Genome-wide analyses of the rice pathogen *Fusarium* *fujikuroi* reveal complex regulation of secondary metabolism and novel metabolites. PLOS Pathog 9(6): e1003475 PubMed: 23825955. 23825955 10.1371/journal.ppat.1003475 PMC3694855

[43] Mihlan M, Homann V, Liu TWD, Tudzynski B (2003) AREA directly mediates nitrogen regulation of gibberellin biosynthesis in *Gibberella* *fujikuroi*, but its activity is not affected by NMR. Mol Microbiol 47: 975-991. doi:10.1046/j.1365-2958.2003.03326.x. PubMed: 12581353. 12581353

[44] Schönig B, Brown DW, Oeser B, Tudzynski B (2008) Cross-species hybridization with *Fusarium* *verticillioides* microarrays reveals new insights into *Fusarium* *fujikuroi* nitrogen regulation and the role of AreA and NMR. Eukaryot Cell 7: 1831-1846. doi:10.1128/EC.00130-08. PubMed: 18689524. 18689524 PMC2568065

[45] Andrianopoulos A, Kourambas S, Sharp JA, Davis MA, Hynes MJ (1998) Characterization of the *Aspergillus* *nidulans* *nmrA* gene involved in nitrogen metabolite repression. J Bacteriol 180: 1973-1977. PubMed: 9537404. 9537404 10.1128/jb.180.7.1973-1977.1998 PMC107119

[46] Wagner D, Schmeinck A, Mos M, Morozov IY, Caddick MX et al. (2010) The bZIP transcription factor MeaB mediates nitrogen metabolite repression at specific loci. Eukaryot Cell 9: 1588-1601. doi:10.1128/EC.00146-10. PubMed: 20729292. 20729292 PMC2950422

[47] Teichert S, Schönig B, Richter S, Tudzynski B (2004) Deletion of the *Gibberella* *fujikuroi* glutamine synthetase gene has significant impact on transcriptional control of primary and secondary metabolism. Mol Microbiol 53: 1661-1675. doi:10.1111/j.1365-2958.2004.04243.x. PubMed: 15341646. 15341646

[48] Schönig B, Vogel S, Tudzynski B (2009) Cpc1 mediates cross-pathway control independently of Mbf1 in *Fusarium* *fujikuroi* . Fungal Genet Biol 46: 898-908. doi:10.1016/j.fgb.2009.08.003. PubMed: 19679194. 19679194

[49] Tudzynski B, Homann V, Feng B, Marzluf GA (1999) Isolation, characterization and disruption of the *areA* nitrogen regulatory gene of *Gibberella* *fujikuroi* . Mol Gen Genet 261: 106-114. doi:10.1007/s004380050947. PubMed: 10071216. 10071216

[50] Darken MA, Jensen AL, Shu P (1959) Production of gibberellic acid by fermentation. Appl Environ Microbiol 7: 301–303. PubMed: 13814121. 10.1128/am.7.5.301-303.1959 PMC1057525 13814121

[51] Geissman TA, Verbiscar AJ, Phinney B, Cragg G (1966) Studies on the biosynthesis of gibberellinf from (-)-kaurenoic acid in cultures of *Gibberella* *fujikuroi* . Phytochemistry 5: 933-947. doi:10.1016/S0031-9422(00)82790-9.

[52] Malonek S, Rojas MC, Hedden P, Gaskin P, Hopkins P et al. (2004) he NADPH-cytochrome P450 reductase gene from *Gibberella* *fujikuroi* is essential for gibberellin biosynthesis. J Biol Chem 279: 25075-25084. doi:10.1074/jbc.M308517200. PubMed: 15037621. 15037621

[53] Cenis JL (1992) Rapid extraction of fungal DNA for PCR amplification. Nucleic Acids Res 20: 2380. doi:10.1093/nar/20.9.2380. PubMed: 1594460. 1594460 PMC312363

[54] Doyle JJ (1990) Isolation of plant DNA from fresh tissue. Focus 12: 13-15.

[55] Sambrook J, Fritsch EF, Maniatis T (1989) Molecular cloning: a laboratory manual. Cold Spring Harbor Laboratory Press, Cold Spring Harbor, NY.

[56] Church GM, Gilbert W (1984) Genomic sequencing. Proc Natl Acad Sci U_S_A 81: 1991-1995. doi:10.1073/pnas.81.7.1991. PubMed: 6326095. 6326095 PMC345422

[57] Altschul SF, Gish W, Miller W, Myers W, Lipman DJ (1990) Basic local alignment search tool. J Mol Biol 251: 403-410. PubMed: 2231712. 10.1016/S0022-2836(05)80360-2 2231712

[58] Colot HV, Park G, Turner GE, Ringelberg C, Crew CM (2006) A high-throughput gene knockout procedure for *Neurospora* reveals functions for multiple transcription factors. Proc Natl Acad Sci U_S_A 103: 10352-10357. doi:10.1073/pnas.0601456103. PubMed: 16801547. 16801547 PMC1482798

[59] Wiemann P, Brown DW, Kleigrewe K, Bok JW, Keller NP (2010) FfVel1 and FfLae1, components of a velvet-like complex in *Fusarium* *fujikuroi*, affect differentiation, secondary metabolism and virulence. Mol Microbiol 77: 972-994. PubMed: 20572938. 20572938 10.1111/j.1365-2958.2010.07263.x PMC2989987

[60] Tudzynski B, Mende K, Weltring KM, Kinghorn JR, Unkles SE (1996) The *Gibberella* *fujikuroi* niaD gene encoding nitrate reductase: isolation, sequence, homologous transformation and electrophoretic karyotype location. Microbiology 142: 533-539. doi:10.1099/13500872-142-3-533. PubMed: 8868428. 8868428

[61] Fleißner A, Diamond S, Glass NL (2009) The *Saccharomyces* *cerevisiae* PRM1 homolog in *Neurospora* *crassa* is involved in vegetative and sexual cell fusion events but also has postfertilization functions. Genetics 181: 497-510. PubMed: 19064710. 19064710 10.1534/genetics.108.096149 PMC2644943

[62] Barendse G, Van De Werken P, Takahashi N (1980) High-performance liquid chromatography of gibberellins. J Chromatogr 198: 449-455. doi:10.1016/S0021-9673(00)80514-2.

[63] Pontecorvo G, Roper JA, Hemmons LM, MacDonald KD, Bufton AW (1953) The genetics of *Aspergillus* *nidulans* . Adv Genet 5: 141-238. doi:10.1016/S0065-2660(08)60408-3. PubMed: 13040135. 13040135

[64] Vogel HJ (1956) A convenient growth medium for *Neurospora*. Microbiol Genet. Bulletin 13: 42-46.

[65] Todd RB, Fraser JA, Wong KH, Davis MA, Hynes MJ (2005) Nuclear accumulation of the GATA factor AreA in response to complete nitrogen starvation by regulation of nuclear export. Eukaryot Cell 4: 1646-165366. doi:10.1128/EC.4.10.1646-1653.2005. PubMed: 16215172. 16215172 PMC1265900

[66] Berger H, Basheer A, Böck S, Reyes-Dominguez Y, Dalik T et al. (2008) Dissecting individual steps of nitrogen transcription factor cooperation in the *Aspergillus* *nidulans* nitrate cluster. Mol Microbiol 69: 1385-1398. doi:10.1111/j.1365-2958.2008.06359.x. PubMed: 18673441. 18673441

[67] Altmann F (1992) Determination of amino sugars and amino acids in glycoconjugates using precolumn derivatization with o-phthalaldehyde. Anal Biochem 204: 215-219. doi:10.1016/0003-2697(92)90164-3. PubMed: 1381156. 1381156

[68] Xiao X, Fu YH, Marzluf GA (1995) The negative-acting NMR regulatory protein of *Neurospora* *crassa* binds to and inhibits the DNA-binding activity of the positive-acting nitrogen regulatory protein NIT2. Biochem 34: 8861–8886. doi:10.1021/bi00027a038. PubMed: 7612627. 7612627

[69] Wong KH, Hynes MJ, Todd RB, Davis MA (2007) Transcriptional control of nmrA by the bZIP transcription factor MeaB reveals a new level of nitrogen regulation in *Aspergillus* *nidulans* . Mol Microbiol 66: 534-551. doi:10.1111/j.1365-2958.2007.05940.x. PubMed: 17854403. 17854403

[70] Calderón J, Martínez LM (1993) Regulation of ammonium ion assimilation enzymes in *Neurospora* *crassa* *nit-2* and *ms-5* mutant strains. Biochem Genet 31: 425-439. doi:10.1007/BF00553459. PubMed: 7907211. 7907211

[71] Schinko T, Berger H, Lee W, Gallmetzer A, Pirker K et al. (2010) Transcriptome analysis of nitrate assimilation in *Aspergillus* *nidulans* reveals connections to nitric oxide metabolism. Mol Microbiol 78: 720–738. doi:10.1111/j.1365-2958.2010.07363.x. PubMed: 20969648. 20969648 PMC3020322

[72] Caddick MX, Peters D, Platt A (1994) Nitrogen regulation in fungi. Antonie Van Leeuwenhoek 65: 169-177. doi:10.1007/BF00871943. PubMed: 7847882. 7847882

[73] Teichert S, Rutherford JC, Wottawa M, Heitman J, Tudzynski B (2008) Impact of ammonium permeases MepA, MepB, and MepC on nitrogen-regulated secondary metabolism in *Fusarium* *fujikuroi* . Eukaryot Cell 7: 187-201. 18083831 10.1128/EC.00351-07 PMC2238153

[74] Rodríguez-García A, Sola-Landa A, Apel K, Santos-Beneit F, Martín JF (2009) Phosphate control over nitrogen metabolism in *Streptomyces* *coelicolor*: Direct and indirect negative control of *glnR*, *glnA*, *glnII* and *amtB* expression by the response regulator PhoP. Nucleic Acids Res 37: 3230-3242. doi:10.1093/nar/gkp162. PubMed: 19321498. 19321498 PMC2691820

[75] Dávila G, Sánchez F, Palacios R, Mora J (1978) Genetics and physiology of *Neurospora* *crassa* glutamine auxotrophs. J Bacteriol 134: 693-698. PubMed: 26664. 26664 10.1128/jb.134.3.693-698.1978 PMC222312

[76] Dávila G, Brom S, Mora Y, Palacios R, Mora J (1983) Genetic and biochemical characterization of glutamine synthetase from *Neurospora* *crassa* glutamine auxotrophs and their revertants. J Bacteriol 156: 993-1000. PubMed: 6139363. 6139363 10.1128/jb.156.3.993-1000.1983 PMC217941

[77] Sánchez F, Calva E, Campomanes M, Blanco L, Guzmán J (1980) Heterogeneity of glutamine synthetase polypeptides in *Neurospora* *crassa* . J Biol Chem 255: 2231-2234. PubMed: 6102088. 6102088

[78] Bauer B, Schwienbacher M, Broniszewska M, Israel L, Heesemann J et al. (2010) Characterisation of the CipC-like protein AFUA_5G09330 of the opportunistic human pathogenic mould *Aspergillus* *fumigatus* . Mycoses 53: 296-304. PubMed: 19486301. 19486301 10.1111/j.1439-0507.2009.01718.x

[79] Böhmer M, Colby T, Böhmer C, Bräutigam A, Schmidt J (2007) Proteomic analysis of dimorphic transition in the phytopathogenic fungus *Ustilago* *maydis* . Proteomics 7: 675-685. doi:10.1002/pmic.200600900. PubMed: 17340586. 17340586

[80] Yamashita MM, Almassy RJ, Janson CA, Cascio D, Eisenberg D (1989) Refined atomic model of glutamine synthetase at 3.5 A resolution. J Biol Chem 264: 17681-17690. PubMed: 2572586. 2572586 10.2210/pdb2gls/pdb

[81] Gill HS, Eisenberg D (2001) The crystal structure of phosphinothricin in the active site of glutamine synthetase illuminates the mechanism of enzymatic inhibition. Biochemistry 40: 1903-1912. doi:10.1021/bi002438h. PubMed: 11329256. 11329256

[82] Meti RS, Ambarish S, Khajure V (2011) Enzymes of ammonia assimilation in fungi: an overview. Recent Res. Science Technol 2: 28-38.

[83] Wiemann P, Albermann S, Niehaus EM, Studt L, von Bargen KW et al. (2012) The Sfp-type 4'-phosphopantetheinyl transferase Ppt1 of *Fusarium* *fujikuroi* controls development, secondary metabolism and pathogenicity. PLOS ONE 7: e37519. doi:10.1371/journal.pone.0037519. PubMed: 22662164. 22662164 PMC3360786

[84] Premakumar R, Sorger GJ, Gooden D (1979) Nitrogen metabolite repression of nitrate reductase in *Neurospora* *crassa* . J Bacteriol 137: 1119-1126. PubMed: 155687. 155687 10.1128/jb.137.3.1119-1126.1979 PMC218290

[85] Premakumar R, Sorger GJ, Gooden D (1980) Physiological characterization of a *Neurospora* *crassa* mutant with impaired regulation of nitrate reductase. J Bacteriol 144: 542-551. PubMed: 6107286. 6107286 10.1128/jb.144.2.542-551.1980 PMC294701

[86] Margelis S, D'Souza C, Small AJ, Hynes MJ, Adams TH et al. (2001) Role of glutamine synthetase in nitrogen metabolite repression in *Aspergillus* *nidulans* . J Bacteriol 183: 5826-5833. doi:10.1128/JB.183.20.5826-5833.2001. PubMed: 11566979. 11566979 PMC99658

[87] Fink D, Falke D, Wohlleben W, Engels A (1999) Nitrogen metabolism in *Streptomyces* *coelicolor* A3 (2): Modification of glutamine synthetase I by an adenylyltransferase. Microbiology 145: 2313-2322. PubMed: 10517584. 10517584 10.1099/00221287-145-9-2313

[88] Wray LV Jr., Fisher SH (2010) Functional roles of the conserved Glu304 loop of *Bacillus* *subtilis* glutamine synthetase. J Bacteriol 192: 5018-5025. doi:10.1128/JB.00509-10. PubMed: 20656908. 20656908 PMC2944536

[89] Hernández G, Mora J (1986) Glutamine synthesis regulates sucrose catabolism in *Neurospora* *crassa* . J Gen Microbiol 132: 3315-3323.

[90] Calderón J, Mora J (1989) Glutamine assimilation pathways in *Neurospora* *crassa* growing on glutamine as sole nitrogen and carbon source. J Gen Microbiol 135: 2699-2707. PubMed: 2576659. 2576659 10.1099/00221287-135-10-2699

[91] Espín G, Palacios R, Mora J (1979) Glutamine metabolism in nitrogen-starved conidia of *Neurospora* *crassa* . J Gen Microbiol 115: 159 PubMed: 43352. 10.1099/00221287-115-1-59 43352

[92] Calderón J, Morett E, Mora J (1985) Omega-amidase pathway in the degradation of glutamine in *Neurospora* *crassa* . J Bacteriol 161: 807-809. PubMed: 2857167. 2857167 10.1128/jb.161.2.807-809.1985 PMC214962

[93] Flores-Samaniego B, Olivera H, González A (1993) Glutamine synthesis is a regulatory signal controlling glucose catabolism in *Saccharomyces* *cerevisiae* . J Bacteriol 175: 7705-7706. PubMed: 7902349. 7902349 10.1128/jb.175.23.7705-7706.1993 PMC206930

[94] Commichau FM, Stülke J (2008) Trigger enzymes: bifunctional proteins active in metabolism and in controlling gene expression. Mol Microbiol 67: 692-702. PubMed: 18086213. 18086213 10.1111/j.1365-2958.2007.06071.x

