# Supplementary figures and images for: A Sensing Role of the Glutamine Synthetase in the Nitrogen Regulation Network in Fusarium fujikuroi
Source: PLoS One. 2013 Nov 15;8(11):e80740. doi: 10.1371/journal.pone.0080740 (PMC3829961; doi:10.1371/journal.pone.0080740)

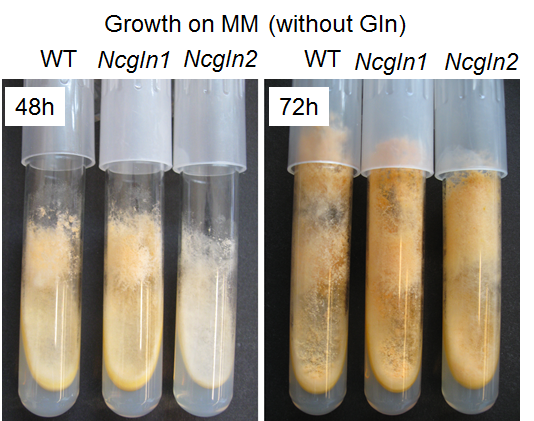

Supplement: Figure S1 — Gln1 and Gln2 have redundant functions in N. crassa. A. Growth of the N. crassa wild type (WT) and the Δgln1 and Δgln2 single mutants on minimal medium (MM) and MM supplemented with glutamine. Maximum linear hyphal extension was determined on race tubes. Error bars indicate standard deviations calculated from three independent experiments. B. Growth of the heterokaryotic double mutant after three days of inoculation compared to the wild type and the single mutants on selective medium (200 μg/ml Nourseothricin) with (top) and without glutamine (bottom). The wild type and the single mutants carried the same resistance marker (NatR), which was used to construct the double mutant. (TIF) [file pone.0080740.s001.tif]
