# Supplementary material for: A Sensing Role of the Glutamine Synthetase in the Nitrogen Regulation Network in Fusarium fujikuroi
Source: PLoS One. 2013 Nov 15;8(11):e80740. doi: 10.1371/journal.pone.0080740 (PMC3829961; doi:10.1371/journal.pone.0080740)
Supplement: Figure S2 — Phylogram of prokaryotic and eukaryotic GS proteins. Protein sequences for the phylogram were retrieved from the Protein Knowledgebase (UniProtKB) by searching for reviewed sequences of glutamine synthetases. Proteins are assigned by Swiss-Prot identifier (sp_). The N. crassa NcGln2 sequence (NCU_06724.5) was retrieved from the Broad Institute Database. Relevant sequences for this study are indicated in bold, the F. fujikuroi sequence in red. (DOCX) [file pone.0080740.s002.docx]

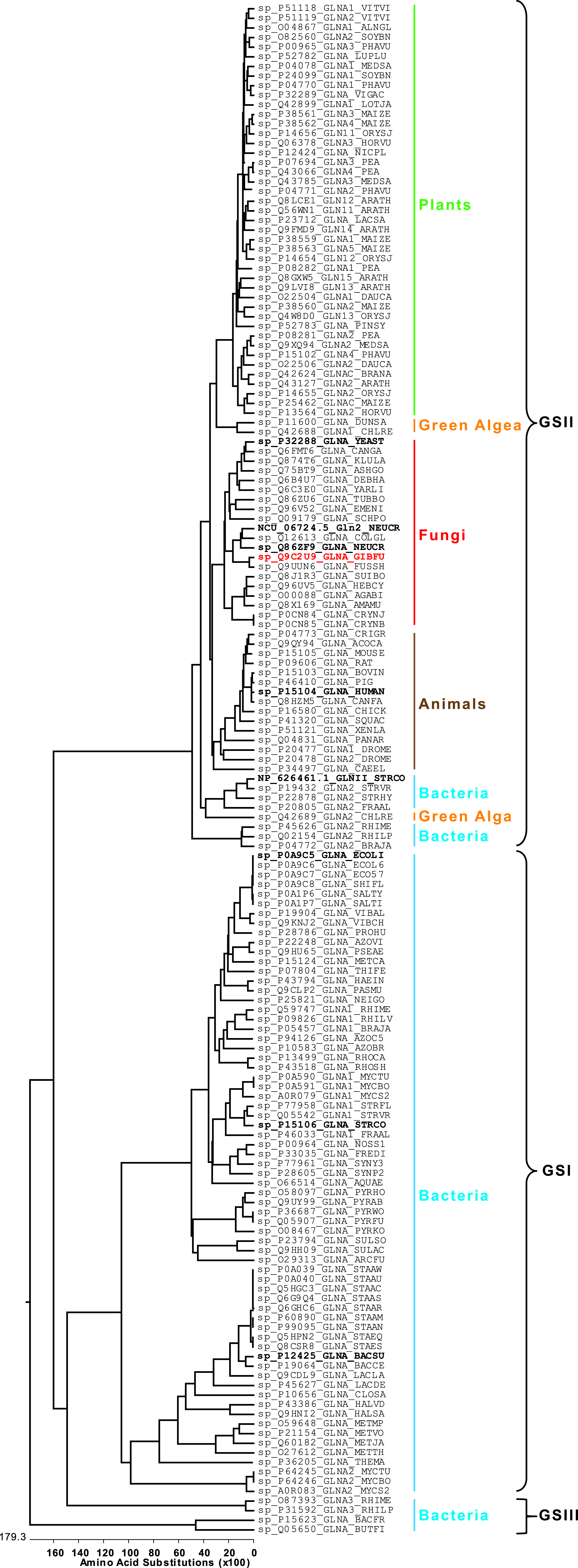


**Figure S2: Phylogram of prokaryotic and eukaryotic GS proteins**

Protein sequences for the phylogram were retrieved from the Protein Knowledgebase (UniProtKB) by searching for reviewed sequences of glutamine synthetases. Proteins are assigned by Swiss-Prot identifier (sp_). The *N. crassa* NcGln2 sequence (NCU_06724.5) was retrieved from the Broad Institute Database. Relevant sequences for this study are indicated in bold, the *F. fujikuroi* sequence in red.
