# Supplementary material for: A Sensing Role of the Glutamine Synthetase in the Nitrogen Regulation Network in Fusarium fujikuroi
Source: PLoS One. 2013 Nov 15;8(11):e80740. doi: 10.1371/journal.pone.0080740 (PMC3829961; doi:10.1371/journal.pone.0080740)
Supplement: Table S1 — Oligonucleotides used in this study. (DOCX) [file pone.0080740.s003.docx]

**Table S1: Oligonucleotides used in this study**

| **Name** | **Sequence** (5‘🡪3‘) | **Purpose** |
| --- | --- | --- |
| ***Fusarium fujikuroi*** | |  |
| GS-D60-S62-F | CGTTTGGAACTTCGCTGGTGCTTCCACTAACCAGGCC | Mutation |
| GS-D60-S62-R | CCTGGTTAGTGGAAGCACCAGCGAAGTTCCAAACGGG | Mutation |
| GS-E130E132-F | AGCCTTGGTTTGGCCTCGCGCAGGCGTATACCCTCCTCGGCTC | Mutation |
| GS-E130E132-R | AGCCGAGGAGGGTATACGCCTGCGCGAGGCCAAACCAAGGCTCG | Mutation |
| GS-E193-F | GATCTCTGGAACGAACGCCGCGGTCATGCCTGCTCAGTGGGAATATCAG | Mutation |
| GS-E193-R | CCCACTGAGCAGGCATGACCGCGGCGTTCGTTCCAGAGATCTGGATGC | Mutation |
| GS-E297-F | cgtttgaccggccgtcacgCgactggatccatcgacagc | Mutation |
| GS-E297-R | gctgtcgatggatccagtcGcgtgacggccggtcaaacg | Mutation |
| GS-e330R332-F | AGGGCTATGGCTACTTTGCGGACTGCCGTCCTGCTTCCAACGCCGATCCC | Mutation |
| GS-e330R332-R | GTTGGAAGCAGGACGGCAGTCCGCAAAGTAGCCATAGCCCTTAGCAGCC | Mutation |
| GS-Ff-F1 | ATCACCTCACGGACGGAGACTCTCC | Diagnostic PCR |
| GS-Ff-R7-Xba | ttgttctctagacgaaacgtgagataaataattgtcggaaagg | Diagnostic PCR |
| GS-G246-G248-F | TGCTGGCGATTGGAACGCAGCTGCTTTGCACTCCAACTTCTCC | Mutation |
| GS-G246-G248-R | GAAGTTGGAGTGCAAAGCAGCTGCGTTCCAATCGCCAGCAATGG | Mutation |
| GS-H250-F | GATTGGAACGGAGCTGGTTTGGCCTCCAACTTCTCCACGAAAGCAATGCG | Mutation |
| GS-H250-R | CATTGCTTTCGTGGAGAAGTTGGAGGCCAAACCAGCTCCGTTCCAATCGC | Mutation |
| GS-H250S254-F | GGAACGGAGCTGGTTTGGCCTCCAACTTCTCCGCGAAAGCAATGC | Mutation |
| GS-H250S254-R | CGCATTGCTTTCGCGGAGAAGTTGGAGGCCAAACCAGCTCCGTTC | Mutation |
| GS-L291-F | ATGGTGAGGATAACGAAGCGCGTTTGACCGGCCGTCACG | Mutation |
| GS-L291-R | CTCGTGACGGCCGGTCAAACGCGCTTCGTTATCCTCACC | Mutation |
| GS-L76-F | CTGGTGACAACTCCGATGTCTACGCCCGTCCCTGCGCCGTCTACCCTTCC | Mutation |
| GS-L76-R | GGGTAGACGGCGCAGGGACGGGCGTAGACATCGGAGTTGTCACCAGGGG | Mutation |
| GS-pDHB1-for | ATTAACAAGGCCATTACGGCCGCTACTCCTATCACCTCACGGACGGAG | Vector cloning |
| GS-pDHB1-for | AACTGATTGGCCGAGGCGGCCCCAGCCATAGAAAATTGGAGGAGAACCTTGGTAAC | Vector cloning |
| GS-pPRN-for | ATTAACAAGGCCATTACGGCCATGGCTACTCCTATCACCTCACGGACG | Vector cloning |
| GS-pPRN-rev | AACTGATTGGCCGAGGCGGCCCTAAGCCATAGAAAATTGGAGGAGAACCTTGGTAA | Vector cloning |
| GS-R311-1-Bam | AGACTGGATCCATCGACAGCTTTTCTTGGGGTGTCGCCCACGGTGGCACAAGCATCC | Mutation |
| GS-R311-2-Bam | AGACTGGATCCATCGACAGCTTTTCTTGGGGTGTCGCCTACTGTGGCACAAGCATCC | Mutation |
| GS-S72-D73-F | CTGGTGACAACGCCGCTGTCTACCTCCGTCCCTGCGCCGTCTACC | Mutation |
| GS-S72-D73-R | gcagggacggaggtagacagcggcgttgtcaccaggggcctgg | Mutation |
| GS-Y159-F | TTCCCTGCTCCCCAAGGCGAGGCCTACTGTGGTGTGGGCACTGG | Mutation |
| GS-Y159-R | CCAGTGCCCACACCACAGTAGGCCTCGCCTTGGGGAGCAGGGAAACC | Mutation |
| niaD-5F | GTAACGCCAGGGTTTTCCCAGTCACGACGTAGGATTCAGTCATCGCATCGC | Gene knock-out |
| niaD-5R | ATCCACTTAACGTTACTGAAATCATGTGTGCCAGTTGTGAAGAGG | Gene knock-out |
| niaD-3F | CTCCTTCAATATCATCTTCTGTCTCTTTGGGCGATGATCTGATGG | Gene knock-out |
| niaD-3R | GCGGATAACAATTTCACACAGGAAACAGCCATAGTGGTGTTGTTCTTGCCC | Gene knock-out |
| niaD-5dia-F | GCAATTACCAAAGCTAACGCCG | Diagnostic PCR |
| niaD-3dia-R | ACTGCGTCAGTTTACTTTCCGC | Diagnostic PCR |
| NiaD-WT-F | AGCCTGAGAAGCAGATCTGC | Diagnostic PCR |
| NiaD-WT-R | AGACGCATCATAAGATGCTGGC | Diagnostic PCR |
| nirA-5F | GTAACGCCAGGGTTTTCCCAGTCACGACGCAAAAGGTGAGGAGCCACATCC | Gene knock-out |
| nirA-5R | ATCCACTTAACGTTACTGAAATCTAACTGAGCAAGCGAAGTCTGG | Gene knock-out |
| nirA-3F | CTCCTTCAATATCATCTTCTGTCGGGCTGAATATTCCCGCTACG | Gene knock-out |
| nirA-3R | GCGGATAACAATTTCACACAGGAAACAGCGGCGATTCAATCACTCCAAGC | Gene knock-out |
| nirA-5DIA-F | GTAGCCAATCTGAGGACTCTCC | Diagnostic PCR |
| nirA-3DIA-R | AGGCTCCAGCATAAGTCAAAGC | Diagnostic PCR |
| nirA-wt-F | TCTGCAGATGTCAAAGCTTTCCG | Diagnostic PCR |
| nirA-wt-R | CACTGCGATGTTACAAATCTGGC | Diagnostic PCR |
| pCSN44-trpC-T | GGAATAGAGTAGATGCCGACCGG | Diagnostic PCR |
| pCSN44-trpC-P | CCTCCACTAGCTCCAGCCAAGCCC | Diagnostic PCR |
| ***Neurospora crassa*** | |  |
| GS-Nc2-F3 | CCAGTAGGCTTGACAAGAGTAATTGTCC | Diagnostic PCR |
| GS-Nc2-R3 | ATCCAAGTTACCGTTCATTCAATAT | Diagnostic PCR |
| GS-Nc2-R1 | TTACTCCGAAACAGCGCCAAAGCAAGTCTCC | Vector cloning |
| GS-Nc1-F2 | ATGTTGGTTTCCAGACCGCACCG | Vector cloning |
| GS-Nc1-R2 | GATAGCCAAGAATCGTCGTCTTCC | Vector cloning |
| GS-Nc1-R-Apa | ATGGGCCCACTGTATCCCGGAACTGTTACGC | Vector cloning |
| GS-Nc1-F-Sal | ATGTCGACAATGGCTACCAAGGAGTCCTTCGCGTCC | Vector cloning |
| ***Streptomyces coelicolor*** | |  |
| GSI-Xho-2-F | ATCTCGAGACAATGTTCCAGAACGCCGACGACGTCAAGAAG | Vector cloning |
| GSII-Xho-F | ATCTCGAGACAATGACCTTCAAGGCCGAGTACATCTGG | Vector cloning |
| GS-Hind-R | ATAAGCTTGAATTCGAGCTCGGTACCCACT | Vector cloning |
| GSI-start-F | GGATGTTCCAGAACGCCGACGACG | Diagnostic PCR |
| GSI-end-R | GATCACACGTCGAAGTACATCTCG | Diagnostic PCR |
| GSII-start-F | CCGGGGTGACCTTCAAGGCC | Diagnostic PCR |
| GSII-end-R | CGCGTCACGACGTACGGGTCCAC | Diagnostic PCR |
