# Supplementary material for: A Sensing Role of the Glutamine Synthetase in the Nitrogen Regulation Network in Fusarium fujikuroi
Source: PLoS One. 2013 Nov 15;8(11):e80740. doi: 10.1371/journal.pone.0080740 (PMC3829961; doi:10.1371/journal.pone.0080740)
Supplement: Table S2 — Identified mutations in the GS-encoding genes Ncgln1 (NCU04856) and Ncgln2 (NCU06724) of mutant strains gln-1a and gln-1b. (DOCX) [file pone.0080740.s004.docx]

**Table S2 Identified mutations in the GS-encoding genes *Ncgln1* (NCU04856) and *Ncgln2* (NCU06724) of mutant strains gln-1a and gln-1b**

| **Mutant Strain** | **gene** | **Mutation** |
| --- | --- | --- |
| *N.crassa*  gln-1a | *Ncgln2* | 1 bp deletion, resulting in a frame shift; early stop codon at  aa 268 |
| *N.crassa*  gln-1a | *Ncgln1* | Prolin for leucin (P130L) |
| *N.crassa*  gln-1b | *Ncgln2* | Phenylalanine for leucine (F221L); glutamate for lysine (E228K) |
| *N.crassa*  gln-1b | *Ncgln1* | Serine for phenylalanine (S6F) |
