# Supplementary material for: A Sensing Role of the Glutamine Synthetase in the Nitrogen Regulation Network in Fusarium fujikuroi
Source: PLoS One. 2013 Nov 15;8(11):e80740. doi: 10.1371/journal.pone.0080740 (PMC3829961; doi:10.1371/journal.pone.0080740)
Supplement: Table S3 — Growth and secondary metabolite production of the F. fujikuroi wild type, the Δgln1 mutant and three complementants carrying site-directed mutations of conserved residues of the F. fujikuroi GS. All strains were grown in minimal medium (MM) with 6 mM or 60 mM glutamine with 440 mM glucose (+C) or without glucose (-C). (DOCX) [file pone.0080740.s005.docx]

**Table S3: Growth and secondary metabolite production of the *F. fujikuroi* wild type, the Δ*gln1* mutant and three complementants carrying site-directed mutations of conserved residues of the *F. fujikuroi* GS. All strains were grown in minimal medium (MM) with 6 mM or 60 mM glutamine with 440 mM glucose (+C) or without glucose (-C)**

| **Strains** | **Growth in MM [mg DW]** | | | | **Production of Bikaverins** | | | | **Production of Gibberellins** | | | |
| --- | --- | --- | --- | --- | --- | --- | --- | --- | --- | --- | --- | --- |
|  | 6 mM | | 60 mM | | 6 mM | | 60 mM | | 6 mM | | 60 mM | |
|  | +C | -C | +C | -C | +C | -C | +C | -C | +C | -C | +C | -C |
| wild type | 1.12 | 0.05 | 1.54 | 0.24 | yes | no | no | no | yes | no | no | no |
| Δ*gln1* | 0.25 | 0.09 | 1.28 | 0.25 | no | no | no | no | no | no | no | no |
| D60A/S62A | 0.69 | 0.07 | 1.26 | 0.29 | yes | no | no | no | yes | no | no | no |
| S72A/D73A | 0.68 | 0.10 | 1.30 | 0.24 | yes | no | no | no | yes | no | no | no |
| G246A/G248A | 0.72 | 0.08 | 1.27 | 0.26 | yes | no | no | no | yes | no | no | no |
